# The Art of Domesticating Proteins: How Cancer Cells Adapt to Therapeutic and Environmental Stressors

**DOI:** 10.3390/ijms27062662

**Published:** 2026-03-14

**Authors:** Slovénie Pyndiah

**Affiliations:** Institut Mondor de Recherche Biomédicale, Faculté de Médecine, Inserm U955, Université Paris-Est Créteil, 94010 Créteil, France; slovenie.pyndiah@u-pec.fr

**Keywords:** drug resistance, protein turnover, targeted therapy, stress adaptation, healthcare

## Abstract

Cellular survival and adaptability depend on the dynamic regulation of proteins—the central actors of biological systems. Through mechanisms such as post-translational modifications, protein turnover, and the formation of membraneless organelles, cells can sense and respond to a variety of stressors. Recent advances in artificial intelligence and chemical biology have provided powerful tools to study and manipulate these processes, paving the way for novel therapeutic strategies in cancer. This review explores how cells “tame” their proteome in response to stress by coordinating protein synthesis, modification, degradation, and structural organization to maintain functional resilience.

## 1. Introduction

Cancer cells maintain their survival through remarkable phenotypic and molecular plasticity, allowing them to adapt to therapeutic, metabolic, and microenvironmental stressors. These adaptive mechanisms operate within tightly regulated spatiotemporal boundaries and are central to treatment outcomes. In oncology, discrepancies between transcript levels and protein abundance are particularly pronounced due to selective protein stabilization, accelerated degradation, and context-dependent translational control [1,2]. As such, the proteome provides the most accurate readout of cancer cell behavior under drug pressure.

Proteins responsible for transport, signalling, DNA repair, and apoptosis are critical determinants of therapeutic responses. Multidrug resistance proteins (e.g., ATP-binding cassette transporters), stress-responsive chaperones, and regulators of cell-cycle checkpoints illustrate how structural and functional protein dynamics shape drug sensitivity [3,4,5]. These dynamics are influenced by post-translational modifications, spatial compartmentalization, and degradation pathways, enabling cancer cells to quickly recalibrate their proteome in response to chemotherapy or targeted inhibitors.

Recent breakthroughs in structural prediction—highlighted by the 2024 Nobel Prize in Chemistry, which recognized artificial intelligence-driven advances in protein structure inference—have transformed our ability to analyze resistance-associated proteins at an unprecedented resolution [6]. Integrating such predictive tools with chemical biology allows for a deeper understanding of conformational shifts, mutational impacts, and allosteric regulation that contribute to drug tolerance.

Against this backdrop, this review reframes the concept of taming the proteome within an oncological context: cancer cells strategically rewire protein synthesis, modification, turnover, and spatial organization to survive therapeutic stress. Understanding these mechanisms provides a foundation for developing drugs that destabilize resistant phenotypes or selectively target the proteins that drive them.

## 2. Protein Turnover: Balancing Synthesis and Degradation in Drug Resistance

### 2.1. Protein Synthesis in Drug Resistance

**Figure 1:**
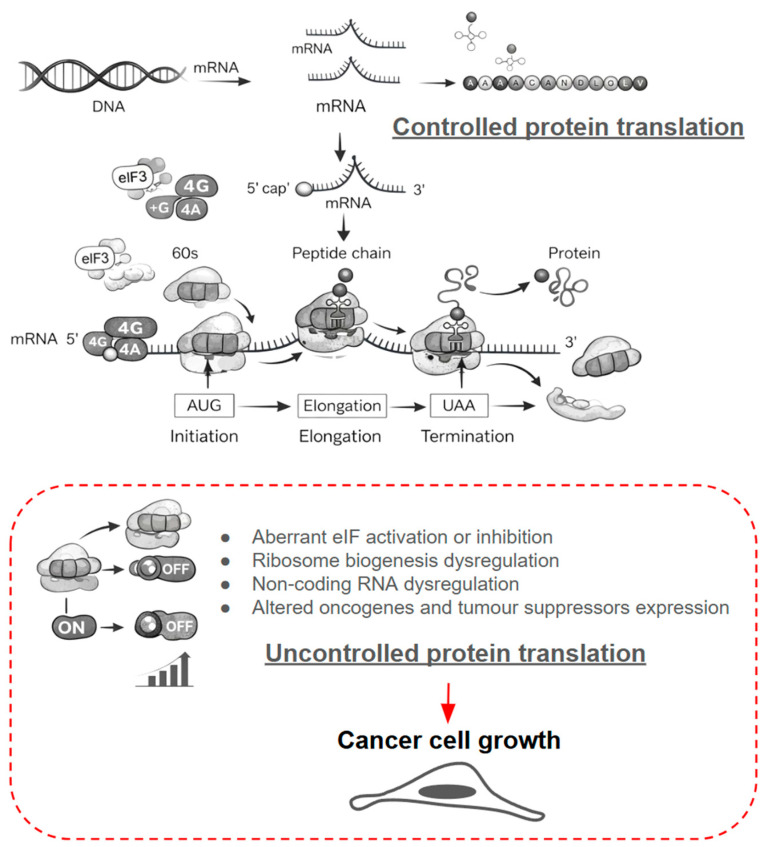
Protein translation mechanisms and the aberrant molecular and cellular phenotypes resulting from their dysregulation [7,8].

Protein turnover begins with synthesis (Figure 1), a finely tuned process that allows cells to remodel their proteome in response to therapeutic pressure. In the context of drug resistance, translational reprogramming enables malignant cells to upregulate efflux pumps, anti-apoptotic proteins, metabolic enzymes, and stress-adaptive factors that counteract drug activity. Because translation initiation is the rate-limiting step, it is the dominant point at which resistant cells modulate protein output [7].

Initiation ensures proper ribosome positioning at the start codon, recruitment of initiator tRNA, and engagement of the elongation cycle. Elongation and termination then drive the stepwise assembly and release of resistance-associated polypeptides. Foundational discoveries defining the genetic code and tRNA structure [9,10], together with advances in ribosome structural biology and mass spectrometry-based proteomics [11,12], have revealed how translational machinery is dynamically rewired under stress, including during chemotherapy, targeted therapy, hypoxia, or nutrient limitation (Figure 1).

Clinically, the translation apparatus represents both a vulnerability and an adaptive tool. Because core elongation and termination functions are essential in all cells, therapeutic strategies largely focus on translation initiation, particularly on eukaryotic initiation factors (eIFs) whose activity governs the expression of survival and resistance genes [7,13]. Pharmacological inhibition of mTOR—one of the central regulators of cap-dependent translation—reduces eIF4E availability through 4E-BP activation, dampening the synthesis of oncogenic drivers recruited during resistance. Clinical evaluation of mTOR pathway inhibition [14], including everolimus in the adjuvant setting for renal cell carcinoma, further illustrates the translational relevance of targeting this axis (Table 1). Experimental modulators such as ribavirin further illustrate how altering eIF activity can disrupt translational programs that support therapeutic escape (Table 1).

Unexpectedly, several antibiotic-derived compounds have revealed additional translational dependencies in drug-resistant cancer cells. Microbial metabolites and their derivatives have emerged as important scaffolds in anticancer drug discovery [42,43]. While classical oxazolidinones selectively inhibit bacterial ribosomes, novel derivatives exhibit anticancer activity in vitro by disrupting stress-responsive translation pathways utilized by rapidly dividing tumour cells. Similarly, macrolide antibiotics such as rapamycin (sirolimus) and its semisynthetic derivatives—temsirolimus and everolimus, collectively known as rapalogs (Table 1)—originate from Streptomyces species and exert anticancer effects by inhibiting mTORC1, a central regulator of protein synthesis and cell growth. Hybrid fluoroquinolone–oxazolidinone scaffolds and related compounds reveal how resistant cancer cells often exploit translational mechanisms similar to microbial stress survival strategies [42,43]. In response to pharmacological stress using microbe-derived compounds, cancer cells adopt molecular adaptations that enhance specific protein functions—such as stronger protein interaction affinities or more efficient DNA repair—contributing to clinically observed drug resistance (Table 2).

When cap-dependent translation is suppressed—whether by therapy-induced stress, hypoxia, or metabolic strain—cells shift to non-canonical initiation [71,72]. Internal ribosome entry site (IRES)-dependent translation allows for continued synthesis of proteins that promote drug resistance [73,74,75], including those regulating the unfolded protein response, angiogenesis, inflammation, apoptosis evasion, and cell-cycle progression. Viral IRES elements have long served as experimental models of this switch [76], revealing mechanisms that resistant cancer cells co-opt (Table 3). IRES-mediated translation is also sensitive to modulation by stress-adaptive drugs (Table 1 and Table 3).

Emerging therapeutics directly target these alternative translation modes. Zotatifin (eFT226), an eIF4A inhibitor in clinical trials (Table 1), suppresses IRES-driven translation of stress-adaptive transcripts required for resistance. Other agents such as JQ1 [IC_50_ = 0.2–10 µM] and CX-5461 [IC_50_ = 0.1–2 µM] indirectly reduce IRES-mediated synthesis of oncogenic drivers like c-Myc [94,95,96], limiting the adaptive proteomic shifts characteristic of resistant tumours (Figure 1, Table 3).

Together, these insights position translational control as a central mechanism through which cells remodel protein abundance during drug exposure. Accordingly, selective targeting of stress-adaptive translation offers a promising route to disrupt resistance-maintaining proteomes (Figure 1, Table 1, Table 2 and Table 3).

### 2.2. Protein Degradation in Drug Resistance

**Figure 2:**
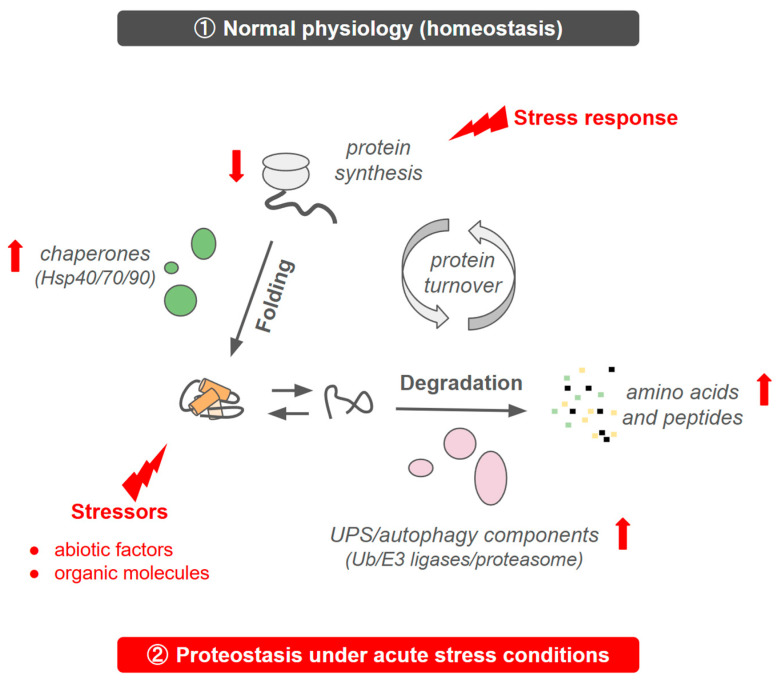
Main pillars of protein turnover in ① normal physiology and ② acute stress conditions, mechanisms that are exploited by cancer cells [97].

Protein degradation constitutes the second major pillar of protein turnover (Figure 2) and plays a decisive role in shaping resistance phenotypes. By eliminating damaged or misfolded proteins and regulating the steady-state abundance of signalling intermediates, the ubiquitin–proteasome system (UPS) and autophagy–lysosome pathway (Figure 2) remodel the proteome during therapeutic stress.

The UPS mediates selective degradation of short-lived or regulatory proteins [98], many of which govern drug sensitivity, cell-cycle control, apoptosis, and DNA damage responses. Through stepwise ubiquitination, substrates are directed to the 26S proteasome for rapid proteolysis, enabling resistant cells to remove pro-apoptotic proteins, suppress negative regulators of survival pathways, or stabilize oncogenic drivers via altered ubiquitin ligase activity [99,100] (Figure 2). Disruption of substrate recognition or proteasome capacity can therefore promote resistance by altering signalling network dynamics. Therapeutic approaches, including proteasome inhibitors and E3 ligase modulators (Table 4), aim to exploit these vulnerabilities by restoring proteostatic imbalance or inducing lethal proteotoxic stress.

In parallel, the autophagy–lysosome system provides a bulk degradation pathway that supports drug-resistant cell survival under metabolic or oxidative stress [141,142]. By clearing damaged organelles and protein aggregates, autophagy preserves mitochondrial function, limits ROS accumulation, and recycles nutrients required for continued proliferation. While impaired autophagic flux can sensitize tumours to therapy, excessive or compensatory autophagy frequently enhances resistance (Figure 2) [141,142], motivating investigation of autophagy modulators as therapeutic adjuncts [99,100].

**Figure 3:**
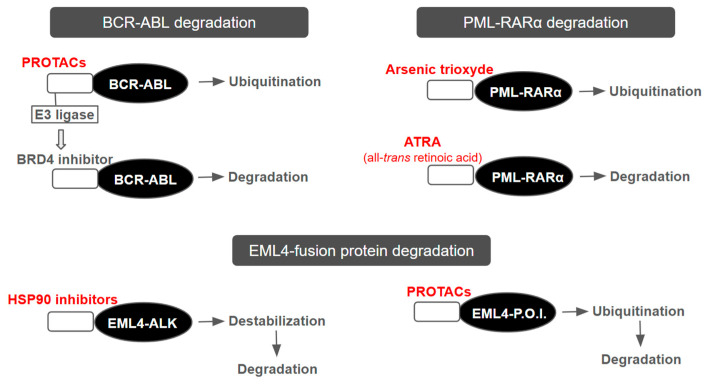
Therapeutic strategies using targeted protein degradation (TPD) [143,144,145].

Targeted Protein Degradation (TPD) technologies further extend these principles by enabling selective elimination of resistance-associated proteins previously considered undruggable [143] (Figure 3). PROTACs and molecular glues recruit endogenous E3 ligases to degrade oncogenic drivers, overcoming limitations of occupancy-based inhibition [99,100,143]. Clinically advanced agents such as ARV-110 and ARV-471 illustrate the therapeutic potential of enforced degradation [99,100].

Importantly, TPD approaches can target oncogenic fusion proteins (e.g., BCR-ABL, PML-RARα, EWS-FLI1) that confer resistance to conventional therapies [99,145,146]. Agents including cereblon-binding molecular glues, arsenic trioxide, ATRA, and HSP90 inhibitors (Table 5) destabilize these aberrant proteins and promote their clearance. By targeting structurally complex or scaffolding-dependent oncoproteins, TPD expands druggability beyond traditional active-site inhibition.

Ultimately, protein degradation provides the rapid adaptive capacity required for resistance evolution. While synthesis gradually builds a resistant proteome (Figure 1), degradative systems enable immediate recalibration of signalling components (Figure 2). However, emerging evidence shows that tumours can also acquire resistance to TPD strategies [148,150,156], highlighting the continued evolutionary plasticity of proteostasis networks.

### 2.3. Clearance and Metabolic Recycling in Drug Resistance

**Figure 4:**
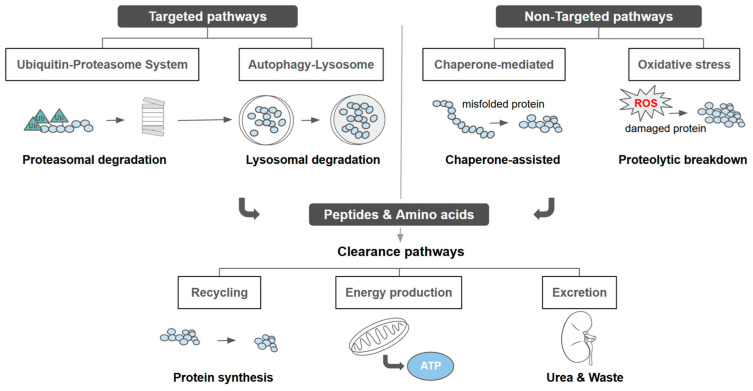
Clearance mechanisms of protein breakdown products through targeted and non-targeted routes.

Clearance and metabolic recycling of protein degradation products are key modulators of cancer drug resistance as they regulate intracellular nutrient availability and detoxification capacity. Anticancer therapies induce proteotoxic stress, increasing proteasomal and lysosomal degradation and generating amino acids and metabolic intermediates that must be efficiently reused or eliminated (Figure 2) [157,158]. As shown in Figure 4, targeted clearance depends on specific amino acid transporters and organelle-associated export systems that channel metabolites toward biosynthesis, signalling, or controlled efflux, whereas non-targeted routes include bulk autophagic turnover and systemic hepatobiliary or renal elimination [159,160].

Lysosomal proteolysis is particularly critical in resistant cancer cells, supplying amino acids that sustain protein synthesis (Figure 1), preserve redox balance, and support mitochondrial function [157]. This recycling limits proteotoxic damage and attenuates therapy-induced apoptosis. Recovered amino acids further activate nutrient-sensing pathways such as mTOR, reinforcing anabolic metabolism under therapeutic stress [158]. Concurrently, resistant cells upregulate amino acid transporters and metabolic enzymes to optimize metabolite uptake and redistribution (Figure 4) [159,161].

Systemic clearance mechanisms additionally shape therapeutic outcomes by regulating xenobiotic and metabolite elimination, thereby influencing intracellular drug accumulation and efficacy [160,161]. Table 6 summarizes clinically used small-molecule inhibitors that target metabolic enzymes, transporters, or clearance pathways. By disrupting amino acid production, utilization, or export, these agents impair metabolic adaptation and enhance treatment sensitivity.

Collectively, intracellular recycling and systemic clearance integrate nutrient recovery with detoxification, enabling cancer cells to maintain bioenergetic stability during therapeutic stress. Targeting these interconnected pathways therefore represents a promising strategy to overcome metabolic resistance. However, tumour cells frequently develop secondary adaptations—including target mutation or amplification, metabolic pathway rewiring, and altered drug transport or efflux (Table 6)—that continue to limit the long-term efficacy of metabolic and antimetabolite therapies used in clinical oncology [180], such as methotrexate, 5-fluorouracil, L-asparaginase, IDH inhibitors, and pentostatin.

## 3. Genotoxic Stressors and the DNA Damage Response in Cancer Therapy

### 3.1. Characteristics of Therapeutically Relevant Genotoxic Stressors

In cancer therapy, genotoxic stressors are intentionally applied to damage tumour DNA and induce cytotoxicity. However, the diversity of DNA lesions they generate—and the tumour cell’s capacity to repair them—creates major challenges in predicting treatment responses [181]. Radiotherapy, chemotherapy, and endogenous sources of DNA damage all contribute to complex mixtures of lesions that activate the DNA damage response (DDR) [182], allowing cancer cells to survive therapy.

Genotoxic agents used in oncology can be broadly categorized into direct and indirect inducers of DNA damage. Direct genotoxicity arises when a therapeutic agent interacts with DNA itself [183], generating chemical or physical lesions that stall replication and transcription. Indirect genotoxicity results from disruptions to cellular metabolism or oxidative balance—such as ROS production (Table 7)—that subsequently injure nuclear DNA [184]. Parameters including chemical reactivity, pharmacokinetics, exposure duration, and intracellular accumulation strongly shape the type and extent of DNA damage generated during treatment [185].

Physical genotoxic agents, particularly those used in radiotherapy [186], generate some of the most cytotoxic lesions in oncology. Ionizing radiation (X-rays, γ-rays, particle beams) produces a spectrum of base damage [187], single-strand breaks (SSBs), and highly lethal double-strand breaks (DSBs), triggering ATM- and ATR-dependent DDR signalling cascades [188]. Non-ionizing UV radiation induces pyrimidine dimers [189] and bulky helix distortions, lesions typically repaired through nucleotide excision repair pathways. Although mechanical or thermal stress contributes less directly to therapeutic DNA damage, they can promote secondary ROS generation [189], amplifying oxidative DNA injury. In clinical practice, radiation exposure in imaging and radiotherapy represents a controlled but potent form of genotoxic stress designed to overwhelm tumour repair capacity [190].

Chemical genotoxic agents (Table 7), including multiple classes of chemotherapeutics, interact covalently or noncovalently with DNA [191]. Alkylating agents (Table 7) introduce alkyl groups onto nucleotide bases, causing mispairing, depurination, helix distortion, and replication fork collapse. Cross-linking agents—such as platinum compounds (Table 7) and nitrogen mustards—form intra- and inter-strand bridges that block both DNA replication and transcription. Attempts by cancer cells to resolve these structures can generate secondary DSBs, making their repair heavily dependent on homologous recombination, Fanconi anemia pathways, and translesion synthesis [192]. These mechanisms are frequently upregulated in therapy-resistant tumours [193].

**Table 7:** Well-characterized DNA-damaging chemicals and the cellular pathways ensuring survival.

Category of DNA Damaging Agents	Mechanism of Action	Common Examples [IC_50_]	Mechanisms Ensuring Cell Survival
Alkylating agents	Covalently attach alkyl groups to DNA bases, causing base mispairing and crosslinking that blocks replication (DNA strand breaks) [194,195]	**Cyclophosphamide** [10–100 µM] **Melphalan** [10–50 µM] **Busulfan** [10–100 µM] **Cisplatin (alkyl-like)** [1–50 µM]	DNA repair upregulation: increased MGMT (O^6^-methylguanine-DNA methyltransferase) expression repairs alkylation damage; enhanced nucleotide excision repair (NER). Drug efflux: overexpression of ABC transporters reduces intracellular drug concentration. Detoxification: elevated glutathione (GSH) and glutathione-S-transferase (GST) activity neutralize electrophiles.
Intercalating agents	Flat aromatic molecules insert between DNA base pairs, distorting the helix and interfering with replication. Causes frameshift mutations, replication inhibition [196,197,198]	**Doxorubicin** [0.1–1 µM] **Daunorubicin** [0.1–10 µM] **Actinomycin D** [0.4–400 nM] **Mitoxantrone** [0.1–1 µM] **Gemcitabine** [0.01–1 µM] **5-Fluorouracil** [0.1–10 µM]	Drug efflux: P-glycoprotein (MDR1/ABCB1) overexpression pumps out intercalators (e.g., doxorubicin, daunorubicin). Topoisomerase alteration: mutation or reduced expression of topoisomerase II reduces drug-target binding. DNA repair: Enhanced homologous recombination or NER repair of DNA adducts.
Reactive oxygen species (ROS), free radicals	Highly reactive oxygen species attack DNA bases and sugars, leading to oxidized bases and single- or double-strand breaks [199,200,201]	**Hydrogen peroxide (H_2_O_2_)** [50–500 µM] **Superoxide (O_2_^−^)** **Hydroxyl radical (•OH)**	Antioxidant defense: upregulation of superoxide dismutase (SOD), catalase, glutathione peroxidase, and glutathione (GSH) to scavenge ROS. Stress response pathways: activation of NRF2/KEAP1 pathway induces cytoprotective genes.
Industrial chemicals (genotoxic)	Various mechanisms: some form DNA adducts, some generate oxidative damage, others interfere with replication enzymes causing chromosomal aberrations (e.g., G/T mutations) [202,203,204,205,206,207]	**Benzene (e.g., benzene oxide, benzo[a]pyrene)** [1–10 µM] **Formaldehyde (CH_2_O)** [50–500 µM] **Vinyl chloride (monomer for PVC)** [50–200 µM] **Per- and polyfluoroalkyl substances (PFASs)** [>10–100 µM]	Metabolic detoxification: increased activity of cytochrome P450 enzymes (e.g., CYP2E1 for benzene leading to benzene oxide). DNA repair: NER, base excision repair (BER), and crosslink repair reducing mutation burden from compounds like benzo[a]pyrene, formaldehyde (CH_2_O), and vinyl chloride. Efflux and sequestration: limited data for PFASs, but cellular sequestration and efflux transporters (e.g., MRP family) may reduce intracellular exposure. Stress-response adaptation: activation of oxidative stress response and phase II detoxifying enzymes such as glutathione S-transferase (GST), NAD(P)H dehydrogenase 1 (NQO1).

Genotoxic stress in human cells can arise not only from therapeutic agents but also from biological sources. Oncoviruses such as Human papillomavirus (HPV), Epstein–Barr virus (EBV), and Hepatitis B virus (HBV) integrate into the host genome or express proteins that inhibit DDR components, including p53 and ATM, thereby promoting mutagenesis and altering therapeutic sensitivity [208]. The bacterial pathogen *Helicobacter pylori*—also classified as a type 1 carcinogen (the highest level) [209]—induces chronic inflammation and ROS-driven DNA damage [210] while fungal metabolites like aflatoxins generate bulky adducts that require nucleotide excision repair [211]. These endogenous and exogenous stressors contribute to replication stress, a driver of genomic instability, cellular senescence, and aging [212]. Chemotherapy itself can exacerbate genotoxic stress in normal tissues, as exemplified by cisplatin-induced DNA lesions and nephrotoxicity [213].

Successfully overcoming drug resistance in cancer demands a multidisciplinary approach, leveraging insights from biology, medicine, physical sciences, mathematics, and data analytics to model DDR dynamics and predict treatment outcomes [214,215]. Long-term chemotherapy also impacts normal hematopoietic cells, highlighting the need to balance tumour targeting with preservation of tissue integrity [216]. Collectively, these biological and therapeutic genotoxic stressors underscore the critical importance of robust, adaptable DDR systems in human cells (Table 7).

### 3.2. DNA Damage, Repair Capacity, and the Biology of Tumour Survival

DNA damage is both a source of oncogenic transformation and a persistent threat to tumour survival. To cope with endogenous lesions and therapy-induced insults, cells rely on the DDR, a coordinated network that integrates DNA repair with cell-cycle checkpoints and global stress responses (Table 7) [217]. DDR genes are frequently mutated in cancer, fostering genomic instability—an enabling hallmark that promotes tumour evolution, metastasis and therapeutic adaptation [218]. Yet these same defects create exploitable vulnerabilities.

A paradigm is provided by BRCA1/2-mutant tumours, which are defective in homologous recombination and therefore hypersensitive to PARP inhibition through synthetic lethality [217,219]. The clinical success of PARP inhibitors validated DDR targeting but also revealed challenges, including resistance mechanisms and the need for predictive biomarkers [219]. Notably, our previous study demonstrated that cisplatin-resistant cancers frequently develop an addiction to PARP1 activity and rely on ATM-mediated DDR signalling by suppressing tumour suppressors [220,221]. Targeting these acquired dependencies establishes a new synthetic lethal framework beyond BRCA mutations, potentially expanding PARP-based therapies to platinum-refractory cancers. Current strategies expand beyond first-generation PARP inhibitors to include next-generation PARP1-selective agents, checkpoint kinase inhibitors and compounds targeting additional “caretaker” repair pathways [217,218]. Simultaneous targeting of multiple DDR nodes or rational combinations with chemo- and radiotherapy are under active investigation [219].

Tumour repair capacity is further shaped by replication stress and transcription-associated DNA damage. Transcription–replication conflicts generate double-strand breaks (DSBs) and genomic rearrangements (Table 7) [222]. Pharmacological targeting of proliferating cell nuclear antigen (PCNA) with AOH1996 selectively exacerbates these conflicts, inducing transcription-dependent DSBs and suppressing tumour growth, thereby exploiting a cancer-selective vulnerability (Table 7) [223].

Beyond enzymatic repair pathways, genome stability depends on nuclear architecture and chromatin dynamics. DSB repair involves chromatin remodelling, repair condensates and large-scale genome reorganization (Figure 2) [224]. Irradiation-induced strengthening of topologically associating domain boundaries in an ATM-dependent manner suggests that 3D genome organization actively contributes to repair fidelity [225]. Moreover, chromatin-modifying complexes such as genetic suppressor element 1 (GSE1), which interacts with a deacetylase/demethylase co-repressor complex, regulate ATR signalling and histone ubiquitination during DDR [226].

Finally, oncogenic mutations rewire protein–protein interaction networks that govern DDR signalling hubs, altering repair pathway choice and stress adaptation (Figure 3) [227,228]. Therapeutic innovation now also leverages platinum–protein interactions to overcome drug efflux and chemoresistance (Table 7) [229]. Collectively, tumour survival reflects a dynamic equilibrium between DNA damage burden, adaptive repair capacity and evolving interaction networks—an equilibrium increasingly amenable to precise therapeutic disruption (Table 7).

## 4. Post-Translational Modifications: A Regulatory Layer in Resistance Acquisition

### 4.1. Ubiquitination in Therapy-Induced Adaptation

**Figure 5:**
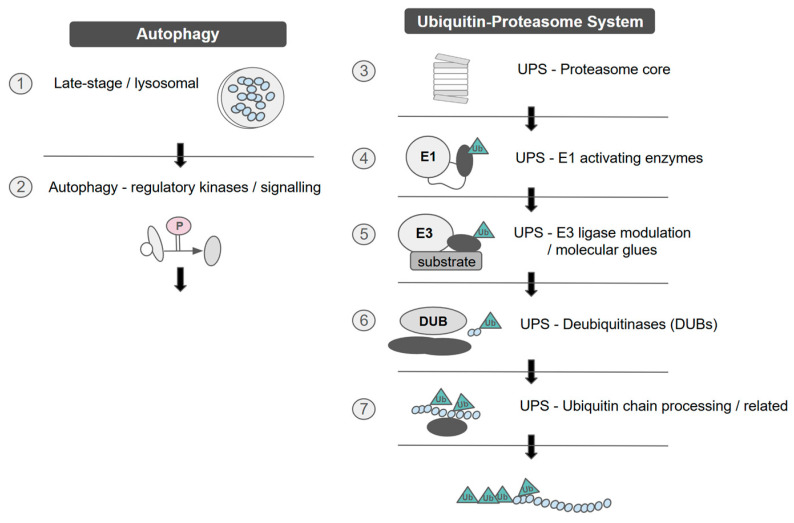
Critical regulatory steps of autophagy and ubiquitin–proteasome system (UPS) targeted by clinically approved small-molecule inhibitors (as mentioned in Table 4).

Ubiquitination, a pivotal post-translational modification, plays a central role in dynamically regulating protein stability and cellular adaptation under therapeutic stress [230]. This process involves the covalent attachment of the 76-amino-acid protein ubiquitin to substrates, either as monoubiquitin or polyubiquitin chains (Figure 4 and Figure 5), orchestrated by the sequential activity of E1, E2, and over 600 E3 ligases [231,232]. Polyubiquitin chains generally signal proteasomal degradation (Figure 2), facilitating rapid clearance of damaged or drug-bound proteins, whereas monoubiquitination modulates non-degradative processes such as protein trafficking, DNA repair, and activation of bypass signalling pathways [233]. The reversibility of ubiquitination, mediated by about 90 human deubiquitinating enzymes (DUBs), allows for precise remodelling of the proteome in response to therapy.

Several components of the ubiquitination machinery, including E1 activating enzymes, E3 ligases, DUBs, and the proteasome itself (Figure 5), have been successfully targeted by small-molecule inhibitors in clinical development or practice (Table 4).

The ubiquitin-proteasome system (UPS) is responsible for the majority of intracellular protein degradation in mammalian cells, yet recent studies indicate that proteasome activity itself, not just rates of ubiquitination, critically determines substrate fate [234]. The 26S proteasome employs a multistep, ATP-dependent mechanism, with structural and biochemical features that ensure selective degradation, efficient ubiquitin recycling, and prevention of non-specific proteolysis (Figure 4). Proteasome function is further regulated by interacting proteins, subunit modifications such as phosphorylation, and coordinated activity with ubiquitinating and deubiquitinating enzymes.

In cancer, dysregulation of ubiquitination or proteasome activity underlies adaptive resistance (Table 4). Altered E3 ligase or DUB expression selectively eliminates pro-apoptotic proteins while stabilizing survival-promoting factors [230,235]. Small-molecule inhibitors, PROTACs, and molecular glues can manipulate this system to degrade oncogenic proteins and overcome resistance, as demonstrated in colorectal cancer and HPV-driven malignancies [230,232]. Collectively, ubiquitination and proteasome regulation constitute a versatile and finely tunable layer of therapy-induced adaptation, offering compelling targets for improving treatment outcomes.

### 4.2. Phosphorylation as a Driver of Adaptive Signalling and Drug Resistance

Post-translational modifications (PTMs) constitute a dynamic regulatory layer that enables cancer cells to rapidly adapt to therapeutic stress. Among these, phosphorylation is particularly central to resistance acquisition because it directly rewires signalling networks without requiring genetic alteration. A global structural analysis of phosphoproteins demonstrated that phosphorylation often induces subtle but stabilizing conformational changes, modulates residue fluctuations, and, in some cases, mechanically couples distal regulatory and functional sites, consistent with allosteric “domino” models of signal propagation [236]. Such structural plasticity provides a mechanistic basis for adaptive signalling in drug-treated cells.

The clinical relevance of phosphorylation as a therapeutic target is reflected in the development of multiple classes of protein kinase inhibitors designed to suppress aberrant oncogenic signalling. The three main classes of protein kinase inhibitors currently used in clinical oncology are summarized in Table 8, highlighting their mechanisms of action.

At the systems level, phosphorylation responses are not uniformly proportional to pathway activation. Quantitative phosphoproteomics of the ERK cascade revealed variable phosphorylation thresholds among ERK substrates, with some sites saturating at low ERK activity and others responding only at high activation levels [242]. Low-threshold targets included transcriptional repressors that promote proliferation when inactivated, whereas high-threshold targets were enriched in DNA damage response proteins. These findings suggest that graded ERK activity can selectively engage proliferative or stress-response programs, thereby shaping cell fate decisions under therapeutic pressure.

Clinically, such adaptive rewiring underlies resistance to kinase inhibitors. Resistance to protein tyrosine kinase inhibitors frequently involves compensatory activation of parallel pathways, microenvironmental cues, and metabolic reprogramming [243]. In TP53-mutated ovarian cancer, a transmembrane receptor-tyrosine-kinase-like orphan receptor 1 (ROR1)–PI3K/AKT signalling node toggles between proliferative bypass and DNA repair states in response to cell cycle blockade [244]. Previously, we showed that cells adapt to Epstein–Barr virus (EBV) infection not only by tolerating translational disruption, but by converting it into a growth-stimulatory signal through selective activation of a PI3K isoform predominantly expressed in B cells [245]. EBV thereby exploits an intrinsic translation–growth feedback loop to promote survival and proliferation of infected cells while simultaneously facilitating immune evasion. Key phosphorylation-dependent resistance mechanisms and associated kinase or signalling inhibitors are detailed in Table 9.

Together, these studies highlight phosphorylation as a tunable, structurally encoded, and threshold-sensitive mechanism that enables dynamic resistance to targeted therapies.

### 4.3. PTMs as Regulators of the DNA Damage Response in Resistant Tumours

PTMs are central regulators of the DDR, particularly in therapy-resistant tumours where proteome plasticity (Figure 2) enables survival under genotoxic stress. PTMs expand protein functional diversity by altering conformation, localization, stability, charge, and protein–protein interactions, thereby rewiring signalling networks that govern DNA repair and cell fate [257]. More than 400–650 PTM types have been described, including phosphorylation, ubiquitination, SUMOylation, acetylation, and redox modifications, many of which dynamically coordinate DDR signalling cascades [257,258].

A key mechanism through which PTMs regulate DDR is through control of protein stability (Figure 2). PTM-regulated degrons determine whether repair factors are stabilized or targeted for proteasomal degradation, fine-tuning the amplitude and duration of repair signalling [259]. In resistant tumours, aberrant phosphorylation or ubiquitination can stabilize oncogenic DDR mediators or inactivate negative regulators, promoting tolerance to chemotherapy or radiotherapy. Large-scale proteogenomic analyses across cancers reveal shared PTM patterns linked to dysregulated DNA repair, particularly phosphorylation-driven DDR subtypes that transcend tissue origin [260].

Beyond histones, non-histone PTMs of checkpoint kinases, repair enzymes, and replication factors critically drive cancer progression and therapeutic resistance [261]. These modifications often occur within multiprotein complexes whose architecture and interaction networks must be carefully represented in mechanistic schematics to capture PTM-dependent regulation [262]. Moreover, molecular chaperones such as HSP90 and HSP70 modulate the stability and PTM state of DDR client proteins, integrating proteostasis (Figure 2) with repair signalling and contributing to treatment resistance when overexpressed [263]. Due to their key role in stabilizing DDR mediators, molecular chaperones have become therapeutic targets (Table 10); however, despite extensive clinical investigation of small-molecule inhibitors, few have achieved regulatory approval as chaperone-specific therapies.

**Figure 6:**
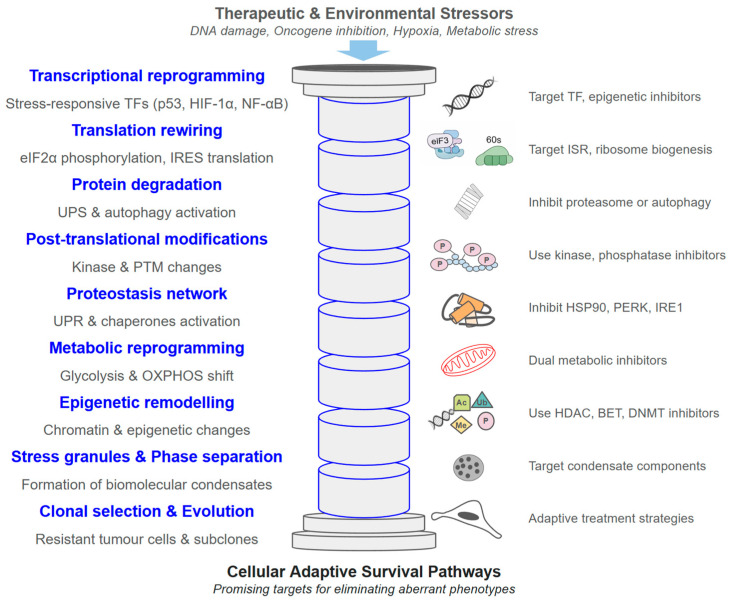
Architectures of proteome remodelling under anthropogenic stress—a perspective inspired by the King’s Garden of Versailles.

Overall, PTMs create a multilayered regulatory system—through cellular signalling pathways, epigenetic modifications, and/or genetic alterations—that allows cancer cells to dynamically integrate genotoxic cues, metabolic state, and therapeutic pressure. Understanding these modification networks (Figure 6) is essential for predicting resistance trajectories and developing next-generation therapies that disrupt the adaptive signalling landscape.

## 5. New Perspectives on Functional Reprogramming: Biomolecular Condensates and Structural Plasticity in Therapy Resistance

### 5.1. Membraneless Organelles and Liquid–Liquid Phase Separation in Treatment Adaptation

Membraneless organelles (MLOs) formed through liquid–liquid phase separation (LLPS) have emerged as central regulators of cellular adaptation to stress and therapy. LLPS enables proteins and RNAs to demix from the surrounding cytoplasm or nucleoplasm into dynamic biomolecular condensates, enriching specific factors while excluding others [272]. These condensates, including stress granules, P-bodies, and signalling hubs, provide rapid and reversible compartmentalization without membrane boundaries, thereby facilitating adaptive responses to environmental and pharmacological stress [273]. In cancer, such plasticity supports treatment adaptation by reorganizing signalling networks, modulating DNA repair foci, and buffering proteotoxic stress (Figure 2 and Figure 6).

**Figure 7:**
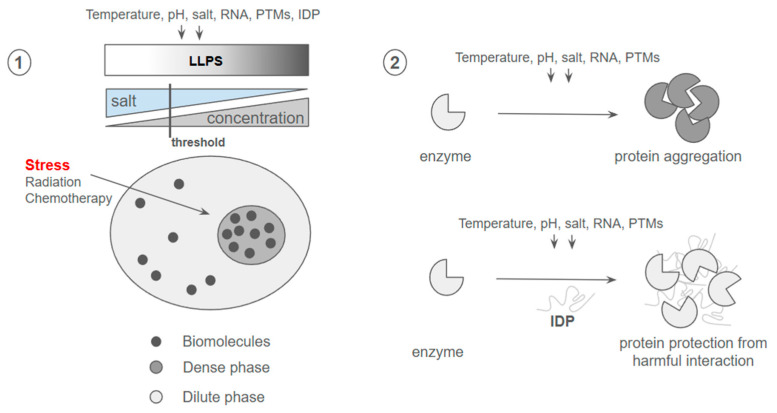
Dynamic condensates formed by ① liquid–liquid phase separation (LLPS) and ② the structural landscape of intrinsically disordered proteins (IDPs) [276,277].

LLPS is governed by multivalent weak interactions influenced by protein concentration, PTMs, pH, ionic strength, and the presence of RNA or partner proteins [274]. Intrinsically disordered regions (IDRs), which lack stable tertiary structures, are particularly important drivers of LLPS. Their high solvent accessibility and conformational flexibility render them sensitive to cellular physicochemistry, enabling them to function as sensors and actuators of stress signals [275]. PTMs within IDRs can shift interaction valency and phase behavior, dynamically tuning condensate assembly during therapeutic challenge [275], shaping the formation of reversible liquid-like condensates or more rigid assemblies (Figure 7).

RNA is not merely a scaffold but an active regulator of condensate dynamics. The updated RPS 2.0 database catalogs over 170,000 LLPS-associated RNAs across 24 condensate types, highlighting the extensive post-transcriptional regulation of phase behavior [278]. Dysregulated LLPS can drive pathological solidification of condensates, contributing to disease states including cancer and neurodegeneration [273,274].

Emerging strategies now explore pharmacological targeting of LLPS, either by modulating PTMs, altering physicochemical conditions, or engineering reversible supramolecular assemblies. Notably, molecular motor-driven systems demonstrate externally controllable LLPS, offering conceptual frameworks for adaptive therapeutic materials (Table 9 and Table 10) [279].

### 5.2. Intrinsically Disordered Proteins and AI-Driven Design of Targeted Therapies

Intrinsically disordered proteins (IDPs) and intrinsically disordered regions (IDRs) challenge the classical structure–function paradigm by lacking a single stable three-dimensional fold while remaining highly functional (Figure 7). Instead, they exist as dynamic ensembles of interconverting conformations whose structural biases are encoded by sequence-dependent interactions [280]. Their high solvent accessibility and conformational plasticity render IDRs exquisitely sensitive to physicochemical changes, positioning them as molecular sensors and actuators of cellular state (Figure 2 and Figure 6) [275]. This responsiveness underlies key roles in signalling, transcription, DNA repair, and stress adaptation—processes frequently dysregulated in cancer and other diseases.

Historically, the structural heterogeneity of IDPs limited their therapeutic tractability. However, advances in computational modelling and artificial intelligence (AI) are transforming this landscape. A general algorithm for designing IDP variants with tailored conformational properties has demonstrated that sequence features can be engineered to modulate compaction, long-range contacts, and phase separation propensity [281]. Integration of machine learning models accelerates prediction of disorder-driven structural behavior, enabling rational tuning of functional properties (Figure 1 and Figure 2).

More recently, generative AI approaches have enabled the design of high-affinity binders that target disordered proteins directly. Using RFdiffusion, researchers generated protein binders against diverse IDPs and IDRs across multiple conformational states, achieving nanomolar affinities and functional activity in cells [282]. Notably, designed binders disrupted stress granule formation via G3BP1 targeting and inhibited amyloid fibril formation, illustrating the therapeutic potential of targeting dynamic disorder rather than fixed structures (Figure 7).

Together, these advances establish a new framework in which IDRs are not obstacles but programmable targets. By coupling biophysical insights into disorder sensitivity [275] with AI-driven protein design, it is now possible to develop precision therapeutics that modulate dynamic conformational ensembles, opening up innovative avenues for targeting previously “undruggable” proteins.

## 6. Conclusions

Across cancer biology, protein regulation emerges not as a static hierarchy, but as a dynamic craft: the art of domestication in which cells continuously sculpt, restrain, and redeploy their proteome to survive stress. Drug resistance is not merely the failure of inhibition but the success of proteostatic adaptation—where synthesis, modification, degradation, spatial organization, and recycling are orchestrated with remarkable precision. Cancer cells do not passively endure therapeutic pressure; they actively tame their proteins.

Small-molecule inhibitors have become the most deliberate tools in this contest. Beyond simple enzyme blockade, they function as proteome sculptors—altering conformational states, exposing or masking degrons, redirecting post-translational modification networks, and reshaping phase-separated environments. Translation inhibitors, kinase blockers, degraders, and DDR modulators reveal that effective therapy increasingly depends on manipulating protein fate rather than single activities. In this sense, modern pharmacology converges with chemical biology: drugs are no longer just antagonists, but agents that reprogram protein lifecycles.

Strikingly, this domestication is not exclusive to designed therapeutics. Environmental anthropic pollutants—heavy metals, endocrine disruptors, particulates, and industrial chemicals—act as unintended small molecules that chronically perturb proteostasis. By inducing oxidative stress, misfolding, aberrant PTMs, and persistent DNA damage, these exposures precondition cells toward adaptive proteome states, shaping resistance long before therapy begins. Cancer evolution thus unfolds at the intersection of clinical intervention and environmental chemistry, where both curated drugs and accidental pollutants apply selective pressure on protein networks.

Advances in AI-driven structural prediction, ensemble modelling, and disorder-aware design now allow us to see this proteomic choreography with unprecedented clarity. As intrinsically disordered proteins, membraneless organelles, and PTM crosstalk come into focus, the challenge shifts from identifying targets to mastering context.

Ultimately, controlling cancer will depend on our ability to domesticate proteins more skillfully than the tumour itself—anticipating adaptive routes, exploiting proteostatic dependencies, and transforming chemical pressure into durable therapeutic control.

## Figures and Tables

**Table 1:** Targeting translation-initiation factors: small-molecule inhibitors and resistance mechanisms (clinical use *).

Targeted Initiation Factor/Pathway	Small-Molecule Inhibitor [IC_50_]	Mechanism of Action	Cancer Context	Resistance Mechanism
eIF4E	Ribavirin * [1–100 µM] Homoharringtonine (HHT) * [10–200 nM]	A guanosine analog reported to compete with the m^7^G cap and impair eIF4E cap-binding and eIF4F assembly; HHT reduces eIF4E phosphorylation and promotes its degradation via SUMOylation [14,15]	Acute myeloid leukemia (AML), solid tumours	eIF4E overexpression; altered drug uptake/metabolism; compensation via 4E-BP phosphorylation; alternative cap-independent translation [16,17]
	**4EGI-1** [10–25 µM] Ouabain * [15–485 nM]	Disrupts eIF4E–eIF4G interaction, impairing formation of the eIF4F complex; ouabain also affects Na^+^/K^+^-ATPase [14]	Leukemia, breast, lung models	Increased eIF4E levels; cap-independent translation; feedback mTOR activation; narrow therapeutic window; AKT/MAPK compensation; ion-homeostasis adaptation [18,19]
eIF4A (RNA helicase)	**Silvestrol (rocaglate family)** [12–85 nM] Zotatifin (eFT226) * [2–15 nM]	Stabilizes interaction of eIF4A with specific mRNAs, blocking translation of structured 5′UTRs [20]	Hematologic malignancies, triple-negative breast cancer (TNBC), solid tumours	ATP-binding cassette transporter-mediated drug efflux; selection for less structured 5′UTR transcripts [21,22]
	**Hippuristanol** [40–60 nM]	Direct inhibitor of eIF4A helicase activity [23]	Lymphoma, leukemia models	[24]
	**Rocaglamide A** [5–15 nM]	Similar to silvestrol; modulates eIF4A–RNA interactions [20]	Hematologic and solid tumour models	Intrinsic mRNA selectivity limits full suppression; adaptive stress signalling [25,26]
eIF2α phosphorylation pathway	**Salubrinal** [5–50 µM]	Indirectly increases phosphorylated eIF2α by inhibiting its dephosphorylation; reduces global translation initiation	Leukemia, solid tumour models	Tumour adaptation via ATF4/CHOP survival pathways; ISR rewiring [27,28]
eIF2B (guanine nucleotide exchange factor)	**Integrated stress response inhibitor (ISRIB)** [5–600 nM]	Stabilizes active eIF2B complex, counteracting inhibition by eIF2α-P (thus restores initiation rather than inhibiting it) [29]	Experimental oncology	Tumour plasticity; bypass via ATF4-driven survival pathways [30,31,32]
MNK1/2 (kinases that regulate eIF4E)	**CGP 57380** [2–15 µM] Tomivosertib (eFT508) * [1–100 µM] BAY 1143269 * [30–40 nM]	Inhibits MNK1/2, thereby reducing eIF4E phosphorylation at serine-209 and affecting translation of select mRNAs, like oncogenic ones [33,34]	Lymphoma, solid tumours	Compensatory MAPK signalling; feedback activation of upstream ERK/p38 pathways [35,36,37]
eIF4F complex globally	**Torin-1** [2–10 nM] **PP242 (mTOR inhibitors)** [50–500 nM] **Sirolimus (rapamycin)** [10–300 nM] **Temsirolimus** [0.5–1 nM] Everolimus (rapalogs) * [30–40 nM]	Inhibits mTORC1 complex (master autophagy regulator), thereby blocking 4E-BP phosphorylation, sequestering eIF4E and suppressing cap-dependent initiation [34,38]	Breast cancer, renal cell carcinoma (RCC), neuroendocrine tumours (NETs)	mTORC2 activation; PI3K/AKT feedback loops; incomplete 4E-BP activation; metabolic adaptation [39,40,41]

**Table 2:** Antibiotic-derived compounds used in cancer therapy and mechanisms of therapeutic resistance.

Drug/Class [IC_50_]	Type of Antibiotic Origin	Cancer Context	Resistance Mechanism
**Doxorubicin (Adriamycin)** [0.1–1 µM]	Anthracycline antibiotic (Streptomyces)	Breast cancer, lymphomas, sarcomas, many solid tumours [44,45]	Efflux pumps (P-glycoprotein), topoisomerase II mutation, enhanced DNA repair [46,47,48]
**Daunorubicin** [0.1–10 µM]		AML, acute lymphoblastic leukemia (ALL) [43,44]	Efflux pumps, topoisomerase II mutation, DNA repair [49,50]
**Epirubicin** [35–500 nM]		Breast cancer, gastric cancer [44,45]	Efflux pumps, topoisomerase II mutation, DNA repair [51,52,53]
**Idarubicin** [5–10 nM]		AML (standard regimens) [44,45]	Efflux pumps, topoisomerase II mutation, DNA repair [54,55,56]
**Bleomycin** [0.02–500 µM]	Glycopeptide, polypeptide antibiotic (Streptomyces)	Hodgkin lymphoma, testicular cancer, some squamous cancers [43]	DNA repair, bleomycin hydrolase [57,58]
**Mitomycin C** [0.1–20 nM]	Aziridine-containing antibiotic	Gastric cancer, anal cancer, superficial bladder cancer [59]	DNA repair, decreased prodrug activation [60,61]
**Actinomycin D (Dactinomycin)** [0.4–400 nM]	Polypeptide antibiotic	Wilms tumour, rhabdomyosarcoma, gestational trophoblastic neoplasia [62]	Efflux, altered DNA binding [63,64,65]
**Plicamycin (Mithramycin)** [20–30 nM]	Polyketide antibiotic	Rarely used; testicular cancer historically [66]	Efflux, altered DNA binding, reduced target sensitivity [67,68]
**Valrubicin** [0.5–3 µM]	Anthracycline derivative	Bladder cancer (intravesical) [44,45]	Efflux pumps, decreased drug uptake [69,70]

**Table 3:** Selected human genes containing internal ribosome entry site (IRES) elements within their untranslated regions (UTRs), enabling cap-independent translation initiation under certain stress conditions.

Gene	Protein Function	IRES Role in Cancer Resistance
*ATF4*	Activating Transcription Factor 4, involved in stress response, plays a key role in the unfolded protein response (UPR)	Under stress (endoplasmic reticulum stress, amino acid deprivation), cap-dependent translation is suppressed, but IRES allows for ATF4 translation; ATF4 then promotes stress adaptation, antioxidant response, and survival under proteasome inhibition and chemotherapy [77,78]
*VEGF*	Vascular Endothelial Growth Factor, involved in angiogenesis, important in tumour growth, wound healing, and vascularization	*VEGF* mRNA contains an IRES, enabling translation under hypoxia or mTOR inhibition, bypassing cap-dependent inhibition; supports angiogenesis and resistance to anti-angiogenic therapy and chemotherapy [79,80,81]
*FGF2*	Fibroblast Growth Factor 2, involved in cell growth, angiogenesis, wound repair	With multiple upstream AUGs, it is a well-characterized IRES; under stress (particularly under hypoxic conditions) or translational suppression, IRES allows for FGF2 translation to promote angiogenesis and therapy resistance [82]
*HIF1A*	Hypoxia-Inducible Factor 1 Alpha, regulates gene expression during low oxygen conditions	HIF1A translation can be IRES-mediated under hypoxia, allowing for continued expression of survival and angiogenesis genes even when cap-dependent translation is inhibited by stress or chemotherapy [83,84]
*c-Fos*	Proto-oncogene involved in cell proliferation, differentiation, and survival, key transcription factor in cancer	Under stress or growth factor conditions, IRES-mediated translation allows for AP-1 component production even when global translation is downregulated; supports survival and proliferation under therapy [85,86]
*c-Myc*	Proto-oncogene that regulates cell cycle progression and apoptosis, important regulator in cancer biology	c-Myc has a well-studied IRES in its 5′ UTR. During stress (mTOR inhibition, chemotherapy), IRES allows for c-Myc translation, maintaining cell growth and proliferation, contributing to therapy resistance [87,88,89]
*eIF4G*	Eukaryotic Initiation Factor 4G, a key component of translation initiation, required for cap-independent translation initiation under various cellular stress conditions	eIF4G is part of the IRES-*trans* acting complex (ITAFs); cleavage or modification of eIF4G during stress switches translation from cap-dependent to IRES-dependent, allowing for selective translation of survival factors like c-Myc, VEGF, ATF4 [72,90]
*p53*	Tumour suppressor protein that regulates the cell cycle and apoptosis, central to many cellular processes, including cancer suppression	*p53* mRNA can contain an IRES, allowing for translation under DNA damage or ribosomal stress conditions and maintaining tumour suppressor functions; mutant p53 can alter IRES usage, contributing to resistance by bypassing apoptosis [91,92,93]

**Table 4:** Protein degradation pathway inhibitors in cancer therapy: clinical use and resistance mechanisms.

Drug/Small Molecule [IC_50_]	Molecular Target	Mechanism of Action	Cancer Context	Resistance Mechanism
**Bortezomib** [5–20 nM]	20S catalytic core of proteasome	Reversible proteasome inhibition, accumulation of misfolded proteins leads to apoptosis	Multiple myeloma, mantle cell lymphoma [101]	Upregulation of proteasome subunits; mutations in β5 subunit (PSMB5); increased drug efflux (MDR-1/P-gp); activation of survival or autophagy pathways [102,103]
**Carfilzomib** [0.01–25 µM]	20S catalytic core of proteasome (β5 irreversible)	Irreversible proteasome inhibition leads to strong proteotoxic stress	Multiple myeloma [104]	Similar mechanisms to bortezomib; MDR-1/P-gp overexpression in resistant models; cross-resistance with other proteasome inhibitors observed [105,106]
**Ixazomib** [3–200 nM]	20S catalytic core of proteasome	Oral proteasome inhibitor, reversible	Multiple myeloma [107,108]	Proteasome subunit overexpression and altered proteasome activity; autophagy induction can support survival [109,110]
**Marizomib** [0.02–2 µM]	20S catalytic core of proteasome (broad inhibition)	Irreversible, it blocks β5, β2, β1 activities	Clinical trials: glioma, multiple myeloma [111,112]	Proteasome subunit alterations; bone marrow microenvironment support; compensatory degradation pathways [113,114]
**Oprozomib** [50–100 nM]	20S catalytic core of proteasome	Oral analog of carfilzomib	Clinical trials for hematologic cancers [115]	Susceptible to MDR-1/P-gp-mediated efflux and adaptive survival; uses mechanisms like autophagy [100,116]
**MLN4924 (Pevonedistat)** [100–400 nM]	NEDD8-activating enzyme (NAE) of the UPS—ubiquitin chain processing	Blocks cullin-RING ligase activation, thereby inhibiting ubiquitination	Phase II trials: acute myeloid leukemia, myelodysplastic syndromes, solid tumours [117,118]	Mutations in NAE/UBA3 prevent drug-NAE adduct formation; compensatory ubiquitin signalling changes may diminish efficacy [119,120,121]
**TAK-243 (MLN7243)** [1–100 nM]	Ubiquitin activating enzyme 1 (UAE1), ubiquitin like modifier activating enzyme 1 (UBA1)	Inhibits UPS—E1 activating enzymes pathway, thereby blocking all ubiquitination	Clinical trials: leukemia, solid tumours [122]	Overexpression of ABC transporters (e.g., ABCB1) reduces intracellular levels; adaptive ubiquitination pathway shifts may counter UBA1 inhibition [123,124]
**Chloroquine, Hydroxychloroquine** [25–100 µM]	Lysosome acidification (autophagy—late-stage/lysosomal)	Blocks autophagosome–lysosome fusion	Clinical use and oncology trials (autophagy inhibition) [125]	Transcriptional plasticity enables cells to bypass autophagy dependence; upregulation of pro-survival pathways; changes in stress response genes [125]
**Arsenic trioxide** [5–50 µM]	Promyelocytic leukemia-retinoic acid receptor α (PML-RARα) fusion protein via SUMOylation and ubiquitination	Induces oncoprotein degradation	Acute promyelocytic leukemia [126,127]	Alterations in oxidative stress handling, metalloid detoxification pathways, and apoptosis regulation can reduce sensitivity (mechanism varies widely by cancer type) [128,129,130]
**Selinexor** [15–500 nM]	Exportin-1 (XPO1)	Traps tumour suppressors in nucleus, thereby enhancing proteotoxic stress	Multiple myeloma, lymphoma [131,132]	Upregulation of alternate nuclear export pathways; changes in XPO1 cargo recognition. Activation of compensatory survival signalling (e.g., NF-κB) [133,134]
**Lenalidomide** [10–50 µM] **Pomalidomide** [1–100 µM]	Cereblon (CRBN), E3 ligase	Induce degradation of Ikaros Family Zinc Finger proteins 1 and 3 (IKZF1/3), neomorphic substrates	Myeloma, lymphoma [135]	Loss-of-function mutations in CRBN or IKZF1/3; alterations in downstream signalling (ERK, microenvironment cues) [136,137,138]
**Iberdomide (CC-220)** [10–150 nM]	CRBN	Next-generation cereblon modulator leads to stronger IKZF1/3 degradation	Clinical trials [139]	Resistance may occur via CRBN/target mutations similar to immunomodulatory imide drugs (IMiDs), but less frequent due to higher CRBN binding affinity [139,140]

**Table 5:** Therapeutic agents promoting degradation of fusion proteins in clinical practice.

Agent Type [IC_50_]	Target Fusion Protein	Mechanism of Action	Cancer Context	Resistance Mechanism
**PROTACs** **(Proteolysis-targeting chimeras)** [1–1000 nM]	BCR-ABL, BRD4 fusions	Recruit E3 ligase leading to ubiquitin-mediated degradation [146]	Hormone-dependent cancers (e.g., prostate cancer: androgen receptor degraders; breast cancer: estrogen receptor degraders), hematologic malignancies, and solid tumours under clinical investigation	Loss, mutation, or downregulation of E3 ligase (e.g., CRBN, VHL) reduces target ubiquitination and degradation; target protein mutation or post-translational modifications can also block PROTAC binding [147,148]
**Arsenic trioxide** [0.5–2 µM]	PML-RARα	SUMOylation leads to ubiquitin-mediated degradation [126,149]	Acute promyelocytic leukemia (APL), especially in combination with ATRA	Increased expression of multidrug resistance transporters (e.g., MRP1/ABCC1), enhanced glutathione synthesis and arsenic detoxification, reducing intracellular accumulation [150]
**ATRA** **(All-*trans* retinoic acid)** [0.01–10 µM]	PML-RARα	Conformational change, thereby inducing proteasomal degradation [151,152]	Acute promyelocytic leukemia (APL), differentiation therapy	Mutations or downregulation of RARα impair receptor-mediated transcription; increased expression of cytochrome P450 (CYP26) enzymes enhances retinoic acid catabolism, lowering effective intracellular levels [153]
**HSP90 inhibitors** [0.002–15 µM]	EML4-ALK	Destabilize HSP90-dependent fusion protein [154,155]	Investigational use in breast cancer, lung cancer, leukemia, and other solid tumours	Upregulation of heat shock proteins (HSP70/HSP27) compensates for HSP90 inhibition; overexpression of drug efflux transporters (e.g., P-glycoprotein) reduces intracellular drug concentration; mutations in the HSP90 ATP-binding domain can also decrease inhibitor binding [156]

**Table 6:** Small-molecule inhibitors targeting cancer metabolism in clinical oncology: mechanisms of action and resistance.

Target Metabolite or Pathway	Target Metabolic Enzyme	Small Molecule Inhibitor [IC_50_]	Mechanism of Action	Cancer Context	Resistance Mechanism
Purine nucleotides	Adenosine deaminase (ADA)	**Pentostatin** [1–2 nM]	Inhibits ADA, leading to toxic deoxyadenosine accumulation in lymphocytes	Hairy cell leukemia [162]	Altered ADA reduces pentostatin sensitivity; upregulation of purine salvage pathways can bypass de novo inhibition [163]
Purine synthesis	Inosine monophosphate dehydrogenase (IMPDH)	**Mycophenolate mofetil** [20–40 nM]	Blocks guanine nucleotide synthesis	Occasionally in lymphoma, transplant-related malignancies [164]	Increased IMPDH expression or flux through salvage pathways restores nucleotide pools [165]
Pyrimidine synthesis	Dihydroorotate dehydrogenase (DHODH)	**Leflunomide** [100–300 nM]	Inhibits de novo pyrimidine synthesis	Investigational in leukemia [166]	Upregulation of DHODH or nucleotide salvage pathways reduces drug efficacy [167]
Folate metabolism	Dihydrofolate reductase (DHFR)	**Methotrexate** [50–100 nM]	Blocks tetrahydrofolate (THF) regeneration leading to a decrease in purine and thymidylate synthesis	Leukemia, lymphoma, osteosarcoma [168,169]	DHFR amplification or reduced uptake via reduced folate carrier (RFC) decreases intracellular drug levels [170]
Thymidylate synthesis	Thymidylate synthase (TS)	**5-Fluorouracil (5-FU)** [5–10 µM]	Inhibits deoxythymidine monophosphate (dTMP) production	Gastrointestinal, breast, head and neck cancers [171]	Increased TS or enhanced nucleotide salvage compensates for inhibition [171]
Asparagine (amino acid depletion)	Asparagine synthetase (indirect targeting)	**L-Asparaginase** [1–10 IU/mL]	Depletes circulating asparagine leading to leukemic cell death	Acute lymphoblastic leukemia [172]	Upregulation of asparagine synthetase restores endogenous asparagine supply [173]
IDH mutant metabolite (2-hydroxyglutarate)	Isocitrate dehydrogenase 1 mutant (IDH1-mutant)	**Ivosidenib (AG-120)** [5–20 nM]	Blocks production of oncometabolite 2-hydroxyglutarate (2-HG)	Acute myeloid leukemia (IDH1-mutant) [174]	Isoform switching (IDH1 ⇆ IDH2) restores 2-HG production [175]
IDH mutant metabolite (2-hydroxyglutarate)	Isocitrate dehydrogenase 2 mutant (IDH2-mutant)	**Enasidenib (AG-221)** [10–100 nM]	Reduces 2-hydroxyglutarate levels	Acute myeloid leukemia (IDH2-mutant) [176,177]	Second-site IDH mutations prevent inhibitor binding; isoform switching (IDH1 ⇆ IDH2) restores 2-HG production [178]
Glutamine metabolism	Glutaminase	**Telaglenastat (CB-839)** [20–30 nM]	Blocks glutamine, leading to glutamate conversion	Investigational (RCC) [179]	Upregulation of glutamine transporters or alternative anaplerotic pathways reduces sensitivity to glutaminase inhibition [179]

**Table 8:** Three main classes of protein kinase inhibitors used in clinics to target overactive kinases in cancer.

Drug Class	Therapeutic Target	Mechanism of Action	Selected Compounds [IC_50_]
Tyrosine kinase inhibitors (TKIs) [237,238]	Receptor tyrosine kinases (RTKs) like EGFR, VEGFR, BCR-ABL	Inhibit ATP-binding site, prevents phosphorylation of downstream signalling proteins	**Imatinib (BCR-ABL)** [0.1–3 µM] **Erlotinib (EGFR)** [2–25 µM] **Crizotinib (ALK)** [0.01–1 µM]
Serine/threonine kinase inhibitors [239]	mTOR, CDKs, MEK	Halt cell cycle or survival signalling, often used in DNA damage response or cell cycle targeting	**Palbociclib (CDK4/6)** [10–20 nM] **Rapamycin** [10–300 nM] **Everolimus (mTOR)** [30–40 nM] **Trametinib (MEK1/2)** [0.5–1 nM] **ATM/ATR inhibitors** [0.001–30 µM]
Multi-kinase inhibitors (MKIs) [240,241]	Multiple kinases (usually tyrosine kinases)	Often >10–20 meaningful targets; inhibit several signalling pathways driving tumour growth; commonly used in RCC, thyroid cancer, hepatocellular carcinoma (HCC)	**Cabozantinib** [0.01–1 nM] **Sunitinib** [0.007–15 µM] **Sorafenib** [20–100 nM] **Lenvatinib** [4–50 nM]

**Table 9:** Targeting phosphorylation in cancer therapy: common kinase and signalling inhibitors in drug resistance.

Drug Class	Representative Anticancer Drugs [IC_50_]	Targeted PTM	Mechanism of Cancer Therapy Failure
Kinase inhibitors	**Imatinib** [0.1–3 µM] **Erlotinib** [2–25 µM] **Sorafenib** [1–10 µM]	Phosphorylation	On-target kinase domain mutations (e.g., gatekeeper mutations such as EGFR T790M, BCR-ABL T315I) reduce drug binding; target amplification increases oncogenic signalling output; activation of bypass pathways (e.g., MET, SRC, PI3K) restores MAPK/AKT signalling; downstream reactivation via RAS/MEK mutations; phenotypic plasticity (EMT, lineage switching such as small-cell transformation); drug efflux transporter upregulation (ABCB1) [246]
CDK inhibitors	**Palbociclib** [10–20 nM] **Ribociclib** [0.1–1 µM] **Abemaciclib** [0.02–0.5 µM]	Phosphorylation	Loss or mutation of RB1 leads to bypass cell-cycle arrest; compensatory activation of CDK2/Cyclin E supports Rb phosphorylation; up-regulation of parallel mitogenic pathways (PI3K/AKT/mTOR, MAPK); reduced drug binding to CDK4/6 conformations [247,248]
mTOR/PI3K inhibitors	**Idelalisib** [0.1–2 µM] **Alpelisib** [0.05–5 µM] **Everolimus** [30–40 nM] **Temsirolimus** [0.5–1 nM]	Phosphorylation	Feedback reactivation of PI3K/AKT signalling upon mTORC1 inhibition; loss of negative regulators (e.g., PTEN) leads to persistent AKT signalling; mutations/altered expression of downstream effectors (S6K1, 4E-BP1) reduces dependence on mTOR; metabolic rewiring and autophagy up-regulation [249,250]
Histone deacetylase (HDAC) inhibitors	**Vorinostat** [0.5–5 µM] **Panobinostat** [5–50 nM] **Romidepsin** [2–20 nM]	Acetylation	Induction or up-regulation of cytoprotective autophagy limits apoptotic cell death; cellular stress response (e.g., NRF2-mediated) alters survival pathways; epigenetic adaptation alters transcriptional programs, reducing drug sensitivity [251]
Proteasome inhibitors	**Bortezomib** [5–20 nM] **Carfilzomib** [0.01–25 µM] **Ixazomib** [3–200 nM]	Ubiquitination (indirectly)	Mutations of proteasome catalytic subunits (e.g., PSMB5) lower drug binding; up-regulation of proteasome subunit expression and alternative proteasome complexes; activation of compensatory stress responses (anti-apoptotic pathways, autophagy); epigenetic and non-mutational drug-tolerant states [116,252]
HSP90 inhibitors	**Ganetespib** [10–100 nM] **Tanespimycin (17-AAG)** [50–500 nM]	Protein folding/stability (chaperone-related PTMs)	Up-regulation of HSP70/HSP90 family as compensatory chaperones; lack of isoform specificity causing suboptimal target engagement and toxicity; activation of alternative client-stabilizing mechanisms [253,254]
PARP inhibitors	**Olaparib** [0.5–5 µM] **Niraparib** [0.15–5 µM] **Talazoparib** [5–50 nM]	Poly-ADP-ribosylation	Restoration of homologous recombination repair (HRR) (e.g., via BRCA1/2 reversion mutations or up-regulation of HR factors); alteration of PARP1 levels or function, reducing inhibition/trapping; protection of replication forks or suppression of DNA damage gaps; increased drug efflux or pharmacokinetic resistance [255,256]

**Table 10:** Targeting the human chaperome: common inhibitors and resistance pathways.

Human Chaperone Protein (Family)	Function/Role	Chaperone Inhibitor [IC_50_]	Resistance Mechanism
HSP90 (HSPC family)	ATP-dependent folding of signalling proteins, kinases, steroid receptors; central proteostasis hub	**17-AAG (Tanespimycin)** [20–100 nM], N-terminal ATPase inhibitor	Upregulation of compensatory heat shock response (HSF1 activation leads to HSP70/HSP27 induction); mutations or conformational changes in HSP90 reduce inhibitor binding; increased drug efflux (e.g., ABC transporters); altered NQO1 expression affecting 17-AAG bioactivation; client protein rewiring/pathway bypass activation [264]
		**17-DMAG (Alvespimycin)** [10–50 nM], more soluble analogue of 17-AAG	Similar to 17-AAG: HSF1-mediated feedback induction; efflux transporter upregulation; adaptive kinase signalling rewiring [264]
		**Ganetespib (STA-9090)** [10–50 nM], potent HSP90 inhibitor	Heat shock response activation; compensatory PI3K/AKT or MAPK pathway activation; reduced drug accumulation [264]
		**Luminespib (AUY922)** [10–100 nM], HSP90 N-terminal inhibitor	HSP70 overexpression; adaptive survival signalling; tumour microenvironment-mediated resistance [265]
		**Onalespib (AT13387)** [10–100 nM], long-acting HSP90 inhibitor	Induction of anti-apoptotic proteins; stress-response reprogramming [264]
		**Others: KW-2478** [50–100 nM], **Debio 0932** [10–50 nM], **PU-H71** [10–50 nM], **BIIB021** [50–100 nM], **TAS-116** [10–50 nM] selective for HSP90α/β	Class-wide mechanisms: HSF1-driven feedback loop, client kinase redundancy, metabolic adaptation [264]
HSP70 (HSPA family)	Folding/refolding of nascent and misfolded proteins; anti-apoptotic roles	**Arimoclomol** [1–10 µM], indirect amplifier of heat shock response	Limited classical resistance described; potential adaptive stress tolerance via broader proteostasis remodelling [266]
		**2-Phenyl ethane sulfonamide (PES)**/**pifithrin-µ** [5–20 µM], disrupts HSP70–client interactions (experimental modulator)	Upregulation of parallel chaperones (HSP90, small HSPs); compensatory autophagy activation; apoptosis pathway adaptation [264]
HSP60 (HSPD family)	Mitochondrial chaperonin; folding of mitochondrial proteins	**Epolactaene/ETB** [1–10 µM] (preclinical)	Mitochondrial stress adaptation; metabolic reprogramming; enhanced mitophagy [267]
Small HSPs (HSPB family)	ATP-independent holdase preventing aggregation; cytoprotection	**KRIBB3** [5–20 µM], affects HSP27 oligomerization and phosphorylation (experimental)	Redundant chaperone compensation; increased anti-apoptotic signalling; altered phosphorylation state of HSP27 [268,269]
Co-chaperones (CDC37, HOP, p23)	Regulate client specificity and chaperone cycle dynamics	**Protein–protein interaction (PPI) disruptors**, dependent on specific chaperone–co-chaperone target (experimental)	Network plasticity; alternative co-chaperone recruitment; client stabilization through parallel pathways [270]
Heat Shock Factor 1 (HSF1)	Master transcriptional regulator of heat shock proteins	**HSF1A**, pathway modulator (experimental)	Transcriptional rewiring; activation of alternative stress-responsive transcription factors; epigenetic adaptation [271]

## Data Availability

No new data were created or analyzed in this study. Data sharing is not applicable to this article.

## Abbreviations

4E-BP—eIF4E-binding protein; ABC—ATP-binding cassette (transporters); AKT—Protein kinase B; ALL—Acute lymphoblastic leukemia; AML—Acute myeloid leukemia; AP-1—Activator protein 1; ATF4—Activating transcription factor 4; ATM—Ataxia telangiectasia mutated; ATR—Ataxia telangiectasia and Rad3-related; BCR-ABL—Breakpoint Cluster Region gene–Abelson murine leukemia viral oncogene homolog 1 fusion; BCR-ABL T315I—Substitution of isoleucine for threonine at codon 315 of BCR-ABL; BER—Base excision repair; BET—Bromodomain and extraterminal domain family proteins; BRCA1/2—Breast cancer susceptibility genes 1 and 2; BRD4 domain—Bromodomain-containing protein 4; CDK—Cyclin-dependent kinase; CH_2_O—Formaldehyde; c-Myc—Proto-oncogene; CYP26—Cytochrome P450 family 26; CYP2E1—Cytochrome P450 family 2 subfamily E member 1; DDR—DNA damage response; DHFR—Dihydrofolate reductase; DHODH—Dihydroorotate dehydrogenase; DNMT—DNA methyltransferases; DSB—Double-strand break; dTMP—Deoxythymidine monophosphate; EBV—Epstein–Barr virus; EGFR T790M—Substitution of methionine for threonine at codon 790 of epidermal growth factor receptor; eIF—Eukaryotic initiation factor; eIF2α—Eukaryotic initiation factor 2 alpha; eIF2B—Eukaryotic initiation factor 2B; eIF4A—RNA helicase component of eIF4F; eIF4E—Cap-binding protein; eIF4F—Translation initiation complex; eIF4G—Scaffold protein of eIF4F; EMT—Epithelial-to-mesenchymal transition; ERK—Extracellular signal-regulated kinase; EWS-FLTI1—Chimeric protein formed by a tumour-specific 11/22 translocation found in both Ewing’s sarcoma and primitive neuroectodermal tumour; FGF2—Fibroblast growth factor 2; GSH—Glutathione; GST—Glutathione-S-transferase; H_2_O_2_—Hydrogen peroxide; HBV—Hepatitis B virus; HDACs—Histone deacetylases; HHT—Homoharringtonine; HIF1A—Hypoxia-inducible factor 1 alpha; HPV—Human papillomavirus; HSP—Heat shock protein; IDH1—Isocitrate dehydrogenase 1; IDH2—Isocitrate dehydrogenase 2; IDP—Intrinsically disordered protein; IDR—Intrinsically disordered region; IKZF1/3—Ikaros family zinc finger proteins 1 and 3; IMPDH—Inosine monophosphate dehydrogenase; IMiDs—Immunomodulatory imide drugs; IRE1—Inositol-requiring transmembrane kinase/endonuclease; IRES—Internal ribosome entry site; ISR—Integrated stress response; ISRIB—Integrated stress response inhibitor; LLPS—Liquid–liquid phase separation; MAPK—Mitogen-activated protein kinase; MDC1—Mediator of DNA damage checkpoint 1; MDR-1/P-gp—Multidrug resistance protein 1/P-glycoprotein; MET—Mesenchymal–epithelial transition factor; MGMT—O^6^-methylguanine-DNA methyltransferase; MNK1/2—MAP kinase-interacting kinases 1 and 2; mTOR—Mechanistic target of rapamycin; mTORC1/2—mTOR complex 1/2; NAE—NEDD8-activating enzyme; NEDD8—Neural precursor cell expressed, developmentally downregulated 8; NETs—Neuroendocrine tumours; NQO1—NAD(P)H dehydrogenase 1; NRF2—Nuclear factor erythroid 2-related factor 2; O^2−^—Superoxide; PCNA—Proliferating cell nuclear antigen; PERK—PKR-like ER kinase; PFASs—Per- and polyfluoroalkyl substances; PI3K—Phosphoinositide 3-kinase; PML-RARα—Promyelocytic leukemia–retinoic acid receptor alpha fusion; PROTAC—Proteolysis-targeting chimera; PSMB5—Proteasome subunit beta type-5; POI—Protein of interest; PTM—Post-translational modification; PVC—Polyvinyl chloride; RCC—Renal cell carcinoma; RAS—Rat sarcoma virus (proto-oncogene); RB1—Retinoblastoma protein 1; RNF8—Ring finger protein 8; ROR1—Receptor tyrosine kinase-like orphan receptor 1; ROS—Reactive oxygen species; RTKs—Receptor tyrosine kinases; S6K1—Ribosomal protein S6 kinase 1; SOD—Superoxide dismutase; SSB—Single-strand break; SUMO—Small ubiquitin-like modifier; SUMOylation—Post-translational modification by covalent attachment of small ubiquitin-like modifier proteins; TF—Transcription factor; TKIs—Tyrosine kinase inhibitors; TNBC—Triple-negative breast cancer; TPD—Targeted protein degradation; TS—Thymidylate synthase; UBA1/UAE1—Ubiquitin-like modifier activating enzyme 1/Ubiquitin activating enzyme 1; UPR—Unfolded protein response; UPS—Ubiquitin–proteasome system; VEGF—Vascular endothelial growth factor; VHL—Von Hippel–Lindau tumour suppressor; XR—X-rays/γ-rays/particle beams.

## References

[1] Schubert U., Antón L.C., Gibbs J., Norbury C.C., Yewdell J.W., Bennink J.R. (2000). Rapid degradation of a large fraction of newly synthesized proteins by proteasomes. Nature.

[2] Diamond P.D., Sauer P.V., Holm M., Swanson-Swett C.J., Ferguson L., Bratset N.M., Wienker G.W., Sim J.S., Adams H.K., Kenner L. (2026). Context-dependent translation inhibition as a cancer therapeutic modality. Nat. Commun..

[3] Long J.E., Wongchenko M.J., Nickles D., Chung W.J., Wang B.E., Riegler J., Li J., Li Q., Sandoval W., Eastham-Anderson J. (2019). Therapeutic resistance and susceptibility is shaped by cooperative multi-compartment tumor adaptation. Cell Death Differ..

[4] Senatore E., Rinaldi L., Chiuso F., Bianco A.G., Feliciello A. (2026). Reshaping cancer cell plasticity by P-body dynamics and protein translation. Commun. Biol..

[5] Pytel D., Fromm Longo J. (2025). The Proteostasis Network in Proteinopathies: Mechanisms and Interconnections. Am. J. Pathol..

[6] Jumper J., Evans R., Pritzel A., Green T., Figurnov M., Ronneberger O., Tunyasuvunakool K., Bates R., Žídek A., Potapenko A. (2021). Highly accurate protein structure prediction with AlphaFold. Nature.

[7] Song P., Yang F., Jin H., Wang X. (2021). The regulation of protein translation and its implications for cancer. Signal Transduct. Target. Ther..

[8] Jia X., He X., Huang C., Li J., Dong Z., Liu K. (2024). Protein translation: Biological processes and therapeutic strategies for human diseases. Signal Transduct. Target. Ther..

[9] Nirenberg M.W., Matthaei J.H. (1961). The dependence of cell-free protein synthesis in E. coli upon naturally occurring or synthetic polyribonucleotides. Proc. Natl. Acad. Sci. USA.

[10] Holley R.W., Apgar J., Everett G.A., Madison J.T., Marquisee M., Merrill S.H., Penswick J.R., Zamir A. (1965). STRUCTURE OF A RIBONUCLEIC ACID. Science.

[11] Fenn J.B., Mann M., Meng C.K., Wong S.F., Whitehouse C.M. (1989). Electrospray ionization for mass spectrometry of large biomolecules. Science.

[12] Wimberly B.T., Brodersen D.E., Clemons W.M., Morgan-Warren R.J., Carter A.P., Vonrhein C., Hartsch T., Ramakrishnan V. (2000). Structure of the 30S ribosomal subunit. Nature.

[13] Sonenberg N., Hinnebusch A.G. (2009). Regulation of translation initiation in eukaryotes: Mechanisms and biological targets. Cell.

[14] Kentsis A., Topisirovic I., Culjkovic B., Shao L., Borden K.L. (2004). Ribavirin suppresses eIF4E-mediated oncogenic transformation by physical mimicry of the 7-methyl guanosine mRNA cap. Proc. Natl. Acad. Sci. USA.

[15] Jin J., Jiang D.Z., Mai W.Y., Meng H.T., Qian W.B., Tong H.Y., Huang J., Mao L.P., Tong Y., Wang L. (2006). Homoharringtonine in combination with cytarabine and aclarubicin resulted in high complete remission rate after the first induction therapy in patients with de novo acute myeloid leukemia. Leukemia.

[16] Beaucourt S., Vignuzzi M. (2014). Ribavirin: A drug active against many viruses with multiple effects on virus replication and propagation: Molecular basis of ribavirin resistance. Curr. Opin. Virol..

[17] Li F., Ling Q., Hu C., Wang H., Ye W., Li X., Zhang X., Lin X., Wei W., Huang X. (2022). Characterization of the Newly Established Homoharringtonine-(HHT-) Resistant Cell Lines and Mechanisms of Resistance. J. Oncol..

[18] Descamps G., Gomez-Bougie P., Tamburini J., Green A., Bouscary D., Maïga S., Moreau P., Le Gouill S., Pellat-Deceunynck C., Amiot M. (2012). The cap-translation inhibitor 4EGI-1 induces apoptosis in multiple myeloma through Noxa induction. Br. J. Cancer..

[19] Weidemann H., Sarsour A.D., Brodie C. (2025). Ouabain—A double-edged sword in tumor development and progression? a review of half a century. Front. Physiol..

[20] Ernst J.T., Thompson P.A., Nilewski C., Sprengeler P.A., Sperry S., Packard G., Michels T., Xiang A., Tran C., Wegerski C.J. (2020). Design of Development Candidate eFT226, a First in Class Inhibitor of Eukaryotic Initiation Factor 4A RNA Helicase. J. Med. Chem..

[21] Zhang W., Gong P., Tian Q., Han S., Wang J., He P., Guo Y., Wang G., Chen Q., Huang J. (2022). The eIF4A Inhibitor Silvestrol Blocks the Growth of Human Glioblastoma Cells by Inhibiting AKT/mTOR and ERK1/2 Signaling Pathway. J. Oncol..

[22] Barranco C. (2025). Anticancer effects of zotatifin are mediated by RNA structure remodelling. Nat. Rev. Mol. Cell Biol..

[23] Cencic R., Im Y.K., Naineni S.K., Moustafa-Kamal M., Jovanovic P., Sabourin V., Annis M.G., Robert F., Schmeing T.M., Koromilas A. (2024). A second-generation eIF4A RNA helicase inhibitor exploits translational reprogramming as a vulnerability in triple-negative breast cancer. Proc. Natl. Acad. Sci. USA.

[24] Steinberger J., Shen L., JKiniry S., Naineni S.K., Cencic R., Amiri M., Aboushawareb S.A.E., Chu J., Maïga R.I., Yachnin B.J. (2020). Identification and characterization of hippuristanol-resistant mutants reveals eIF4A1 dependencies within mRNA 5’ leader regions. Nucleic Acids Res..

[25] Luo J., Ng W., Liu Y., Wang L., Gong C., Zhou Y., Fang C., Zhu S., Yao C. (2024). Rocaglamide promotes infiltration and differentiation of T cells and coordinates with PD-1 inhibitor to overcome checkpoint resistance in multiple tumor models. Cancer Immunol. Immunother..

[26] He Q., Schuessler P.J., Srinivasan A., Caprino J., Barbi J., Walker S.E. (2025). Translation Inhibition by Rocaglamide A Enhances Susceptibility of Yeasts to Caspofungin. bioRxiv.

[27] Bryant K.F., Macari E.R., Malik N., Boyce M., Yuan J., Coen D.M. (2008). ICP34.5-dependent and -independent activities of salubrinal in herpes simplex virus-1 infected cells. Virology.

[28] Chen M.C., Hsu L.L., Wang S.F., Pan Y.L., Lo J.F., Yeh T.S., Tseng L.M., Lee H.C. (2021). Salubrinal Enhances Cancer Cell Death during Glucose Deprivation through the Upregulation of xCT and Mitochondrial Oxidative Stress. Biomedicines.

[29] Sekine Y., Zyryanova A., Crespillo-Casado A., Fischer P.M., Harding H.P., Ron D. (2015). Stress responses: Mutations in a translation initiation factor identify the target of a memory-enhancing compound. Science.

[30] Sidrauski C., McGeachy A.M., Ingolia N.T., Walter P. (2015). The small molecule ISRIB reverses the effects of eIF2α phosphorylation on translation and stress granule assembly. elife.

[31] Zyryanova A.F., Weis F., Faille A., Alard A.A., Crespillo-Casado A., Sekine Y., Harding H.P., Allen F., Parts L., Fromont C. (2018). Binding of ISRIB reveals a regulatory site in the nucleotide exchange factor eIF2B. Science.

[32] Hu R., Chen X., Su Q., Wang Z., Wang X., Gong M., Xu M., Le R., Gao Y., Dai P. (2024). ISR inhibition reverses pancreatic β-cell failure in Wolfram syndrome models. Cell Death Differ..

[33] Santag S., Siegel F., Wengner A.M., Lange C., Bömer U., Eis K., Pühler F., Lienau P., Bergemann L., Michels M. (2017). BAY 1143269, a novel MNK1 inhibitor, targets oncogenic protein expression and shows potent anti-tumor activity. Cancer Lett..

[34] Hsieh A.C., Liu Y., Edlind M.P., Ingolia N.T., Janes M.R., Sher A., Shi E.Y., Stumpf C.R., Christensen C., Bonham M.J. (2012). The translational landscape of mTOR signalling steers cancer initiation and metastasis. Nature.

[35] Huang X.B., Yang C.M., Han Q.M., Ye X.J., Lei W., Qian W.B. (2018). MNK1 inhibitor CGP57380 overcomes mTOR inhibitor-induced activation of eIF4E: The mechanism of synergic killing of human T-ALL cells. Acta Pharmacol. Sin..

[36] Wan W., Zhang X., Huang C., Chen L., Yang X., Bao K., Peng T. (2022). Preclinical anti-angiogenic and anti-cancer activities of BAY1143269 in glioblastoma via targeting oncogenic protein expression. Pharmacol. Res. Perspect..

[37] Ferrario C., Mackey J., Gelmon K.A., Levasseur N., Sorensen P.H., Oo H.Z., Negri G.L., Tse V.W.L., Spencer S.E., Cheng G. (2025). Phase Ib Pharmacodynamic Study of the MNK Inhibitor Tomivosertib (eFT508) Combined with Paclitaxel in Patients with Refractory Metastatic Breast Cancer. Clin. Cancer Res..

[38] Ryan C.W., Tangen C.M., Heath E.I., Stein M.N., Meng M.V., Alva A.S., Pal S.K., Puzanov I., Clark J.I., Choueiri T.K. (2023). Adjuvant everolimus after surgery for renal cell carcinoma (EVEREST): A double-blind, placebo-controlled, randomised, phase 3 trial. Lancet.

[39] Lamming D.W. (2016). Inhibition of the Mechanistic Target of Rapamycin (mTOR)-Rapamycin and Beyond. Cold Spring Harb. Perspect. Med..

[40] Rodrik-Outmezguine V.S., Okaniwa M., Yao Z., Novotny C.J., McWhirter C., Banaji A., Won H., Wong W., Berger M., de Stanchina E. (2016). Overcoming mTOR resistance mutations with a new-generation mTOR inhibitor. Nature.

[41] Nakayama Y., Enomoto D., Yamamoto K., Takara K. (2023). Molecular Characteristics of Everolimus-resistant Renal Cell Carcinoma Cells Generated by Continuous Exposure to Everolimus. Anticancer Res..

[42] Kapoor R., Saini A., Sharma D. (2022). Indispensable role of microbes in anticancer drugs and discovery trends. Appl. Microbiol. Biotechnol..

[43] Giurini E.F., Godla A., Gupta K.H. (2024). Redefining bioactive small molecules from microbial metabolites as revolutionary anticancer agents. Cancer Gene Ther..

[44] Mattioli R., Ilari A., Colotti B., Mosca L., Fazi F., Colotti G. (2023). Doxorubicin and other anthracyclines in cancers: Activity, chemoresistance and its overcoming. Mol. Aspects Med..

[45] Chen Y., Yang W., Cui X., Zhang H., Li L., Fu J., Guo H. (2024). Research Progress on the Mechanism, Monitoring, and Prevention of Cardiac Injury Caused by Antineoplastic Drugs-Anthracyclines. Biology.

[46] Giddings E.L., Champagne D.P., Wu M.H., Laffin J.M., Thornton T.M., Valenca-Pereira F., Culp-Hill R., Fortner K.A., Romero N., East J. (2021). Mitochondrial ATP fuels ABC transporter-mediated drug efflux in cancer chemoresistance. Nat. Commun..

[47] Wei L., Kim S.H., Armaly A.M., Aubé J., Xu L., Wu X. (2024). HuR inhibition overcomes cFLIP-mediated doxorubicin resistance in triple-negative breast cancer. npj Precis. Oncol..

[48] Alimohammadi M., Kahkesh S., Khoshnazar S.M., Khajehpour H., Farahani N., Alaei E., Mafi A., Hashemi M., Hedayati N., Taheriazam A. (2025). Circular RNAs and doxorubicin resistance in cancer: Molecular mechanisms and potential treatment targets. Gene.

[49] Codini M., Conte C., Cataldi S., Arcuri C., Lazzarini A., Ceccarini M.R., Patria F., Floridi A., Mecca C., Ambesi-Impiombato F.S. (2018). Nuclear Lipid Microdomains Regulate Daunorubicin Resistance in Hepatoma Cells. Int. J. Mol. Sci..

[50] Kaczorowska A., Sareło P., Gąsior-Głogowska M., Zioło E., Karpiński P., Łaczmański Ł., Sobas M., Wróbel T., Podbielska H., Kopaczyńska M. (2025). Flow cytometry-based targeted diagnostics for rapid assessment of daunorubicin resistance in acute myeloid leukemia. Sci. Rep..

[51] Sayal K., Gounaris I., Basu B., Freeman S., Moyle P., Hosking K., Iddawela M., Jimenez-Linan M., Abraham J., Brenton J. (2015). Epirubicin, Cisplatin, and Capecitabine for Primary Platinum-Resistant or Platinum-Refractory Epithelial Ovarian Cancer: Results of a Retrospective, Single-Institution Study. Int. J. Gynecol. Cancer.

[52] Felipe A.V., Oliveira J., Moraes A.A., França J.P., Silva T.D., Forones N.M. (2018). Reversal of Multidrug Resistance in an Epirubicin-Resistant Gastric Cancer Cell Subline. Asian Pac. J. Cancer Prev..

[53] Wang F., Yang S., Lv M., Chen F., Yin H., Gao S., Tang J., Yu J. (2021). Novel Long Noncoding RNA 005620 Induces Epirubicin Resistance in Triple-Negative Breast Cancer by Regulating ITGB1 Expression. Front. Oncol..

[54] Komiyama T., Ogura A., Kajiwara T., Okada Y., Kobayashi H. (2018). Analysis of Candidate Idarubicin Drug Resistance Genes in MOLT-3 Cells Using Exome Nuclear DNA. Genes..

[55] Zhang Y., Wei W., Li C., Yan S., Wang S., Xiao S., He C., Li J., Qi Z., Li B. (2022). Idarubicin combats abiraterone and enzalutamide resistance in prostate cells via targeting XPA protein. Cell Death Dis..

[56] van Gelder M.A., Li Y., Wander D.P.A., Berlin I., Overkleeft H.S., van der Zanden S.Y., Neefjes J.J.C. (2024). Novel N,N-Dimethyl-idarubicin Analogues Are Effective Cytotoxic Agents for ABCB1-Overexpressing, Doxorubicin-Resistant Cells. J. Med. Chem..

[57] Dortet L., Girlich D., Virlouvet A.L., Poirel L., Nordmann P., Iorga B.I., Naas T. (2017). Characterization of BRPMBL, the Bleomycin Resistance Protein Associated with the Carbapenemase NDM. Antimicrob. Agents Chemother..

[58] Belinfante A., Kolar J., Shore S.M., Nicholson T.L. (2026). The Streptococcus suis ble gene does not encode a functional bleomycin resistance protein. Vet. Microbiol..

[59] Deng T., Liu B., Duan X., Zhang T., Cai C., Zeng G. (2017). Systematic Review and Cumulative Analysis of the Combination of Mitomycin C plus Bacillus Calmette-Guérin (BCG) for Non-Muscle-Invasive Bladder Cancer. Sci. Rep..

[60] Shannon N.B., Tan J.W., Tan H.L., Wang W., Chen Y., Lim H.J., Tan Q.X., Hendrikson J., Ng W.H., Loo L.Y. (2019). A set of molecular markers predicts chemosensitivity to Mitomycin-C following cytoreductive surgery and hyperthermic intraperitoneal chemotherapy for colorectal peritoneal metastasis. Sci. Rep..

[61] Petersen M.E., Khamas A.B., Østergaard L.J., Jørgensen N.P., Meyer R.L. (2025). Combination therapy delays antimicrobial resistance after adaptive laboratory evolution of Staphylococcus aureus. Antimicrob. Agents Chemother..

[62] Tiburcio P.D.B., Chen K., Xu L., Chen K.S. (2024). Actinomycin D and bortezomib disrupt protein homeostasis in Wilms tumor. bioRxiv.

[63] Willson J.K., Long B.H., Marks M.E., Brattain D.E., Wiley J.E., Brattain M.G. (1984). Mitomycin C resistance in a human colon carcinoma cell line associated with cell surface protein alterations. Cancer Res..

[64] Tajer L., Paillart J.C., Dib H., Sabatier J.M., Fajloun Z., Abi Khattar Z. (2024). Molecular Mechanisms of Bacterial Resistance to Antimicrobial Peptides in the Modern Era: An Updated Review. Microorganisms.

[65] Katayama E., Usui H., Nakamura N., Sato A., Sakai N., Otsuka S., Okuya R., Habu Y., Matsuoka A., Nishikimi K. (2025). Comparison of the efficacy and safety of five-day methotrexate versus pulse actinomycin D for low-risk gestational trophoblastic neoplasia: A single-center historical cohort study^☆^. Int. J. Gynecol. Cancer.

[66] Choi E.S., Nam J.S., Jung J.Y., Cho N.P., Cho S.D. (2014). Modulation of specificity protein 1 by mithramycin A as a novel therapeutic strategy for cervical cancer. Sci. Rep..

[67] Nivina A., Grieb M.S., Loot C., Bikard D., Cury J., Shehata L., Bernardes J., Mazel D. (2020). Structure-specific DNA recombination sites: Design, validation, and machine learning-based refinement. Sci. Adv..

[68] Deng J., Tian A.L., Pan H., Sauvat A., Leduc M., Liu P., Zhao L., Zhang S., Chen H., Taly V. (2021). Everolimus and plicamycin specifically target chemoresistant colorectal cancer cells of the CMS4 subtype. Cell Death Dis..

[69] Georgievski A., Bellaye P.S., Tournier B., Choubley H., Pais de Barros J.P., Herbst M., Béduneau A., Callier P., Collin B., Végran F. (2024). Valrubicin-loaded immunoliposomes for specific vesicle-mediated cell death in the treatment of hematological cancers. Cell Death Dis..

[70] Wani M.K., Koseki Y., Yarber R.H., Sweatman T.W., Ahmed A., Samant S., Hengesteg A., Israel M., Robbins K.T. (2000). Rationale for intralesional valrubicin in chemoradiation of squamous cell carcinoma of the head and neck. Laryngoscope.

[71] Solodushko V., Fouty B. (2023). Terminal hairpins improve protein expression in IRES-initiated mRNA in the absence of a cap and polyadenylated tail. Gene Ther..

[72] Mahé M., Rios-Fuller T., Katsara O., Schneider R.J. (2024). Non-canonical mRNA translation initiation in cell stress and cancer. NAR Cancer.

[73] Walters B., Thompson S.R. (2016). Cap-Independent Translational Control of Carcinogenesis. Front. Oncol..

[74] Badawi A., Biyanee A., Nasrullah U., Winslow S., Schmid T., Pfeilschifter J., Eberhardt W. (2018). Inhibition of IRES-dependent translation of caspase-2 by HuR confers chemotherapeutic drug resistance in colon carcinoma cells. Oncotarget.

[75] Damiano F., Di Chiara Stanca B., Giannotti L., Stanca E., Dinoi A.F., Siculella L. (2025). Targeting Cancer Translational Plasticity: IRES-Driven Metabolism and Survival Within the Tumor Microenvironment. Cancers.

[76] Lee K.M., Chen C.J., Shih S.R. (2017). Regulation Mechanisms of Viral IRES-Driven Translation. Trends Microbiol..

[77] Labbé K., LeBon L., King B., Vu N., Stoops E.H., Ly N., Lefebvre A.E.Y.T., Seitzer P., Krishnan S., Heo J.M. (2024). Specific activation of the integrated stress response uncovers regulation of central carbon metabolism and lipid droplet biogenesis. Nat. Commun..

[78] Adjibade P., Mazroui R. (2026). Regulation of Translation of ATF4 mRNA: A Focus on Translation Initiation Factors and RNA-Binding Proteins. Cells.

[79] Cammas A., Dubrac A., Morel B., Lamaa A., Touriol C., Teulade-Fichou M.P., Prats H., Millevoi S. (2015). Stabilization of the G-quadruplex at the VEGF IRES represses cap-independent translation. RNA Biol..

[80] LaFargue C.J., Amero P., Noh K., Mangala L.S., Wen Y., Bayraktar E., Umamaheswaran S., Stur E., Dasari S.K., Ivan C. (2023). Overcoming adaptive resistance to anti-VEGF therapy by targeting CD5L. Nat. Commun..

[81] Lee C., Kim M.J., Kumar A., Lee H.W., Yang Y., Kim Y. (2025). Vascular endothelial growth factor signaling in health and disease: From molecular mechanisms to therapeutic perspectives. Signal Transduct. Target. Ther..

[82] Tétu P., Delyon J., André J., Reger de Moura C., Sabbah M., Ghanem G.E., Battistella M., Mourah S., Lebbé C., Dumaz N. (2020). FGF2 Induces Resistance to Nilotinib through MAPK Pathway Activation in KIT Mutated Melanoma. Cancers.

[83] Yi T., Papadopoulos E., Hagner P.R., Wagner G. (2013). Hypoxia-inducible factor-1α (HIF-1α) promotes cap-dependent translation of selective mRNAs through up-regulating initiation factor eIF4E1 in breast cancer cells under hypoxia conditions. J. Biol. Chem..

[84] Yang Z., Su W., Wei X., Pan Y., Xing M., Niu L., Feng B., Kong W., Ren X., Huang F. (2025). Hypoxia inducible factor-1α drives cancer resistance to cuproptosis. Cancer Cell.

[85] Li H., Chen Y., Zhang J., Lin Y., Yang Z., Tan J., Qiao W. (2021). Identification of the internal ribosome entry sites in the 5’-untranslated region of the c-fos gene. Int. J. Mol. Med..

[86] Kuonen F., Li N.Y., Haensel D., Patel T., Gaddam S., Yerly L., Rieger K., Aasi S., Oro A.E. (2021). c-FOS drives reversible basal to squamous cell carcinoma transition. Cell Rep..

[87] Stoneley M., Subkhankulova T., Le Quesne J.P., Coldwell M.J., Jopling C.L., Belsham G.J., Willis A.E. (2000). Analysis of the c-myc IRES; a potential role for cell-type specific trans-acting factors and the nuclear compartment. Nucleic Acids Res..

[88] Yeh D.W., Zhao X., Siddique H.R., Zheng M., Choi H.Y., Machida T., Narayanan P., Kou Y., Punj V., Tahara S.M. (2023). MSI2 promotes translation of multiple IRES-containing oncogenes and virus to induce self-renewal of tumor initiating stem-like cells. Cell Death Discov..

[89] Casacuberta-Serra S., González-Larreategui Í., Capitán-Leo D., Soucek L. (2024). MYC and KRAS cooperation: From historical challenges to therapeutic opportunities in cancer. Signal Transduct. Target. Ther..

[90] Amiri M., Mahmood N., Tahmasebi S., Sonenberg N. (2025). eIF4F-mediated dysregulation of mRNA translation in cancer. RNA.

[91] Ji B., Harris B.R., Liu Y., Deng Y., Gradilone S.A., Cleary M.P., Liu J., Yang D.Q. (2017). Targeting IRES-Mediated p53 Synthesis for Cancer Diagnosis and Therapeutics. Int. J. Mol. Sci..

[92] Marcel V., Nguyen Van Long F., Diaz J.J. (2018). 40 Years of Research Put p53 in Translation. Cancers.

[93] Fusée L.T.S., Marín M., Fåhraeus R., López I. (2020). Alternative Mechanisms of p53 Action During the Unfolded Protein Response. Cancers.

[94] Deng X., Yu Y.V., Jin Y.N. (2024). Non-canonical translation in cancer: Significance and therapeutic potential of non-canonical ORFs, m6A-modification, and circular RNAs. Cell Death Discov..

[95] Kang J., Brajanovski N., Chan K.T., Xuan J., Pearson R.B., Sanij E. (2021). Ribosomal proteins and human diseases: Molecular mechanisms and targeted therapy. Signal Transduct. Target. Ther..

[96] Lee H.C., Wang H., Baladandayuthapani V., Lin H., He J., Jones R.J., Kuiatse I., Gu D., Wang Z., Ma W. (2017). RNA Polymerase I Inhibition with CX-5461 as a Novel Therapeutic Strategy to Target MYC in Multiple Myeloma. Br. J. Haematol..

[97] Klaips C.L., Jayaraj G.G., Hartl F.U. (2018). Pathways of cellular proteostasis in aging and disease. J. Cell Biol..

[98] Müller L., Hoppe T. (2024). UPS-dependent strategies of protein quality control degradation. Trends Biochem. Sci..

[99] Tsai J.M., Nowak R.P., Ebert B.L., Fischer E.S. (2024). Targeted protein degradation: From mechanisms to clinic. Nat. Rev. Mol. Cell Biol..

[100] Zhong G., Chang X., Xie W., Zhou X. (2024). Targeted protein degradation: Advances in drug discovery and clinical practice. Signal Transduct. Target. Ther..

[101] Sogbein O., Paul P., Umar M., Chaari A., Batuman V., Upadhyay R. (2024). Bortezomib in cancer therapy: Mechanisms, side effects, and future proteasome inhibitors. Life Sci..

[102] Li F., Liu J., Fu Y. (2024). Acquired Bortezomib Resistance in Multiple Myeloma: From Mechanisms to Strategy. Curr. Treat. Options Oncol..

[103] Kozalak G., Bütün İ., Toyran E., Koşar A. (2023). Review on Bortezomib Resistance in Multiple Myeloma and Potential Role of Emerging Technologies. Pharmaceuticals.

[104] Landgren O., Sonneveld P., Jakubowiak A., Mohty M., Iskander K.S., Mezzi K., Siegel D.S. (2019). Carfilzomib with immunomodulatory drugs for the treatment of newly diagnosed multiple myeloma. Leukemia.

[105] Besse A., Stolze S.C., Rasche L., Weinhold N., Morgan G.J., Kraus M., Bader J., Overkleeft H.S., Besse L., Driessen C. (2018). Carfilzomib resistance due to ABCB1/MDR1 overexpression is overcome by nelfinavir and lopinavir in multiple myeloma. Leukemia.

[106] Żyłka K., Łuczak M., Kostrzewska-Poczekaj M., Bednarek K., Bolomsky A., Kubicki T., Jarmuż-Szymczak M., Ludwig H., Dytfeld D. (2025). Carfilzomib resistance in multiple myeloma: A comparative metabolomic analysis. J. Appl. Genet..

[107] Moreau P., Masszi T., Grzasko N., Bahlis N.J., Hansson M., Pour L., Sandhu I., Ganly P., Baker B.W., Jackson S.R. (2016). Oral Ixazomib, Lenalidomide, and Dexamethasone for Multiple Myeloma. N. Engl. J. Med..

[108] Cook G., Ashcroft A.J., Senior E., Olivier C., Hockaday A., Richards J., Cavenagh J.D., Snowden J.A., Drayson M.T., de Tute R. (2024). Ixazomib as consolidation and maintenance versus observation in patients with relapsed multiple myeloma eligible for salvage autologous stem-cell transplantation (Myeloma XII [ACCoRD]): Interim analysis of a multicentre, open-label, randomised, phase 3 trial. Lancet Haematol..

[109] Kumar S., Moreau P., Hari P., Mateos M.V., Ludwig H., Shustik C., Masszi T., Spencer A., Hájek R., Romeril K. (2017). Management of adverse events associated with ixazomib plus lenalidomide/dexamethasone in relapsed/refractory multiple myeloma. Br. J. Haematol..

[110] Bobin A., Manier S., De Keizer J., Srimani J.K., Hulin C., Karlin L., Caillot D., Lafon I., Mariette C., Araujo C. (2025). Ixazomib, pomalidomide and dexamethasone in relapsed or refractory multiple myeloma characterized with highrisk cytogenetics: The IFM 2014-01 study. Haematologica.

[111] Rajan A.M., Kumar S. (2016). New investigational drugs with single-agent activity in multiple myeloma. Blood Cancer J..

[112] Roth P., Gorlia T., Reijneveld J.C., de Vos F., Idbaih A., Frenel J.S., Le Rhun E., Sepulveda J.M., Perry J., Masucci G.L. (2024). Marizomib for patients with newly diagnosed glioblastoma: A randomized phase 3 trial. Neuro Oncol..

[113] Niewerth D., Jansen G., Riethoff L.F., van Meerloo J., Kale A.J., Moore B.S., Assaraf Y.G., Anderl J.L., Zweegman S., Kaspers G.J. (2014). Antileukemic activity and mechanism of drug resistance to the marine Salinispora tropica proteasome inhibitor salinosporamide A (Marizomib). Mol. Pharmacol..

[114] Piskorz W.M., Krętowski R., Cechowska-Pasko M. (2024). Marizomib Promotes Senescence or Long-Term Apoptosis in Melanoma Cancer Cells. Molecules.

[115] Ghobrial I.M., Vij R., Siegel D., Badros A., Kaufman J., Raje N., Jakubowiak A., Savona M.R., Obreja M., Berdeja J.G. (2019). A Phase Ib/II Study of Oprozomib in Patients with Advanced Multiple Myeloma and Waldenström Macroglobulinemia. Clin. Cancer Res..

[116] Bo Kim K. (2021). Proteasomal adaptations to FDA-approved proteasome inhibitors: A potential mechanism for drug resistance?. Cancer Drug Resist..

[117] Tong S., Si Y., Yu H., Zhang L., Xie P., Jiang W. (2017). MLN4924 (Pevonedistat), a protein neddylation inhibitor, suppresses proliferation and migration of human clear cell renal cell carcinoma. Sci. Rep..

[118] Ferris J., Espona-Fiedler M., Hamilton C., Holohan C., Crawford N., McIntyre A.J., Roberts J.Z., Wappett M., McDade S.S., Longley D.B. (2020). Pevonedistat (MLN4924): Mechanism of cell death induction and therapeutic potential in colorectal cancer. Cell Death Discov..

[119] Zheng S., Leclerc G.M., Li B., Swords R.T., Barredo J.C. (2017). Inhibition of the NEDD8 conjugation pathway induces calcium-dependent compensatory activation of the pro-survival MEK/ERK pathway in acute lymphoblastic leukemia. Oncotarget.

[120] Wei L.Y., Wu Z.X., Yang Y., Zhao M., Ma X.Y., Li J.S., Yang D.H., Chen Z.S., Fan Y.F. (2020). Overexpression of ABCG2 confers resistance to pevonedistat, an NAE inhibitor. Exp. Cell Res..

[121] Shoji H., Takahari D., Ooki A., Hirano H., Okita N., Nagashima K., Adachi J., Yamaguchi K., Kato K., Boku N. (2025). Phase I study of pevonedistat combined with capecitabine and oxaliplatin in patients with platinum-refractory advanced gastric cancer. Sci. Rep..

[122] Hyer M.L., Milhollen M.A., Ciavarri J., Fleming P., Traore T., Sappal D., Huck J., Shi J., Gavin J., Brownell J. (2018). A small-molecule inhibitor of the ubiquitin activating enzyme for cancer treatment. Nat. Med..

[123] Boer D.R., Bijlmakers M.J. (2019). Differential Inhibition of Human and Trypanosome Ubiquitin E1S by TAK-243 Offers Possibilities for Parasite Selective Inhibitors. Sci. Rep..

[124] Zhang X., Wu R., Tian C., Wang W., Zhou L., Guo T., Yu J., Wu C., Shen Y., Liu X. (2022). GRP78 blockade overcomes intrinsic resistance to UBA1 inhibitor TAK-243 in glioblastoma. Cell Death Discov..

[125] Vaena S.G., Romeo M.J., Mina-Abouda M., Funk E.C., Fullbright G., Long D.T., Delaney J.R. (2024). Autophagy unrelated transcriptional mechanisms of hydroxychloroquine resistance revealed by integrated multi-omics of evolved cancer cells. Cell Cycle.

[126] Jiang Y., Shen X., Zhi F., Wen Z., Gao Y., Xu J., Yang B., Bai Y. (2023). An overview of arsenic trioxide-involved combined treatment algorithms for leukemia: Basic concepts and clinical implications. Cell Death Discov..

[127] Gill H., Raghupathy R., Hou H.A., Cheng-Hong Tsai X., Tantiworawit A., Ooi M.G., Gan G.G., Wong C.L., Yim R., Chin L. (2025). Acute Promyelocytic Leukemia Asian Consortium study of arsenic trioxide in newly diagnosed patients: Impact and outcome. Blood Adv..

[128] Liu X., Chen J., Yu S., Yan L., Guo H., Dai J., Zhang W., Zhu J. (2017). All-trans retinoic acid and arsenic trioxide fail to derepress the monocytic differentiation driver Irf8 in acute promyelocytic leukemia cells. Cell Death Dis..

[129] Balasundaram N., Ganesan S., Chendamarai E., Palani H.K., Venkatraman A., Alex A.A., David S., Kumar S.P., Radhakrishnan N.R., Yasar M. (2022). Metabolic adaptation drives arsenic trioxide resistance in acute promyelocytic leukemia. Blood Adv..

[130] Yu P.H., Zhu C.Y., Kang Y.Y., Naranmandura H., Yang C. (2025). Mutation in the Unrearranged PML Allele Confers Resistance to Arsenic Trioxide in Acute Promyelocytic Leukemia. Research.

[131] Chari A., Vogl D.T., Gavriatopoulou M., Nooka A.K., Yee A.J., Huff C.A., Moreau P., Dingli D., Cole C., Lonial S. (2019). Oral Selinexor-Dexamethasone for Triple-Class Refractory Multiple Myeloma. N. Engl. J. Med..

[132] Kalakonda N., Maerevoet M., Cavallo F., Follows G., Goy A., Vermaat J.S.P., Casasnovas O., Hamad N., Zijlstra J.M., Bakhshi S. (2020). Selinexor in patients with relapsed or refractory diffuse large B-cell lymphoma (SADAL): A single-arm, multinational, multicentre, open-label, phase 2 trial. Lancet Haematol..

[133] Lin K.H., Rutter J.C., Xie A., Killarney S.T., Vaganay C., Benaksas C., Ling F., Sodaro G., Meslin P.A., Bassil C.F. (2022). P2RY2-AKT activation is a therapeutically actionable consequence of XPO1 inhibition in acute myeloid leukemia. Nat. Cancer.

[134] Zheng X., Wang C., Chen F., Li S., Zhang H., Dong G., Yang S., Kang X., Kang Z., Han C. (2024). Zanubrutinib delays selinexor resistance evolution in biopsy sample-derived primary central nervous system lymphoma models. iScience.

[135] Kulig P., Milczarek S., Bakinowska E., Szalewska L., Baumert B., Machaliński B. (2023). Lenalidomide in Multiple Myeloma: Review of Resistance Mechanisms, Current Treatment Strategies and Future Perspectives. Cancers.

[136] Zhu Y.X., Shi C.X., Bruins L.A., Wang X., Riggs D.L., Porter B., Ahmann J.M., de Campos C.B., Braggio E., Bergsagel P.L. (2019). Identification of lenalidomide resistance pathways in myeloma and targeted resensitization using cereblon replacement, inhibition of STAT3 or targeting of IRF4. Blood Cancer J..

[137] Ng Y.L.D., Ramberger E., Bohl S.R., Dolnik A., Steinebach C., Conrad T., Müller S., Popp O., Kull M., Haji M. (2022). Proteomic profiling reveals CDK6 upregulation as a targetable resistance mechanism for lenalidomide in multiple myeloma. Nat. Commun..

[138] Zhuang Y., Li C., Jiang H., Li L., Zhang Y., Yu W., Fu W. (2023). Multi-omics investigation of the resistance mechanisms of pomalidomide in multiple myeloma. Front. Oncol..

[139] Bjorklund C.C., Kang J., Amatangelo M., Polonskaia A., Katz M., Chiu H., Couto S., Wang M., Ren Y., Ortiz M. (2020). Iberdomide (CC-220) is a potent cereblon E3 ligase modulator with antitumor and immunostimulatory activities in lenalidomide- and pomalidomide-resistant multiple myeloma cells with dysregulated CRBN. Leukemia.

[140] Słabicki M., Park J., Nowak R.P., Roy Burman S.S., Pellman J., Zou C., Razumkov H., Carreiro J., Rastogi S., Goldstein A. (2025). Expanding the druggable zinc-finger proteome defines properties of drug-induced degradation. Mol. Cell.

[141] White E., Lattime E.C., Guo J.Y. (2021). Autophagy Regulates Stress Responses, Metabolism, and Anticancer Immunity. Trends Cancer.

[142] Liu S., Yao S., Yang H., Liu S., Wang Y. (2023). Autophagy: Regulator of cell death. Cell Death Dis..

[143] Yoon H., Rutter J.C., Li Y.D., Ebert B.L. (2024). Induced protein degradation for therapeutics: Past, present, and future. J. Clin. Investig..

[144] Zhao L., Zhao J., Zhong K., Tong A., Jia D. (2022). Targeted protein degradation: Mechanisms, strategies and application. Signal Transduct. Target. Ther..

[145] Liu J., Tokheim C., Lee J.D., Gan W., North B.J., Liu X.S., Pandolfi P.P., Wei W. (2021). Genetic fusions favor tumorigenesis through degron loss in oncogenes. Nat. Commun..

[146] Cai H., Zhang T., Hu Y. (2025). Global landscape of PROTAC: Perspectives from patents, drug pipelines, clinical trials, and licensing transactions. Eur. J. Med. Chem..

[147] Békés M., Langley D.R., Crews C.M. (2022). PROTAC targeted protein degraders: The past is prologue. Nat. Rev. Drug Discov..

[148] Kurimchak A.M., Herrera-Montávez C., Montserrat-Sangrà S., Araiza-Olivera D., Hu J., Neumann-Domer R., Kuruvilla M., Bellacosa A., Testa J.R., Jin J. (2022). The drug efflux pump MDR1 promotes intrinsic and acquired resistance to PROTACs in cancer cells. Sci. Signal..

[149] Chin L., Kumana C.R., Kwong Y.L., Gill H. (2022). The Development and Clinical Applications of Oral Arsenic Trioxide for Acute Promyelocytic Leukaemia and Other Diseases. Pharmaceutics.

[150] Pearson S.A., Cowan J.A. (2021). Glutathione-coordinated metal complexes as substrates for cellular transporters. Metallomics.

[151] Giuli M.V., Hanieh P.N., Giuliani E., Rinaldi F., Marianecci C., Screpanti I., Checquolo S., Carafa M. (2020). Current Trends in ATRA Delivery for Cancer Therapy. Pharmaceutics.

[152] Rehman U.U., Lübbert M. (2025). All-trans retinoic acid beyond acute promyelocytic leukemia. Cancer Cell.

[153] Isoherranen N., Zhong G. (2019). Biochemical and physiological importance of the CYP26 retinoic acid hydroxylases. Pharmacol. Ther..

[154] Li Z.N., Luo Y. (2023). HSP90 inhibitors and cancer: Prospects for use in targeted therapies. Oncol. Rep..

[155] Kim Y., Lim S.Y., Kim H.O., Ha S.J., Park J.A., Won Y.W., Chae S., Lim K.S. (2025). Combination Strategies with HSP90 Inhibitors in Cancer Therapy: Mechanisms, Challenges, and Future Perspectives. Pharmaceuticals.

[156] Vogt M., Dienstbier N., Schliehe-Diecks J., Scharov K., Tu J.W., Gebing P., Hogenkamp J., Bilen B.S., Furlan S., Picard D. (2023). Co-targeting HSP90 alpha and CDK7 overcomes resistance against HSP90 inhibitors in BCR-ABL1+ leukemia cells. Cell Death Dis..

[157] Higuchi-Sanabria R., Shen K., Kelet N., Frankino P.A., Durieux J., Bar-Ziv R., Sing C.N., Garcia E.J., Homentcovschi S., Sanchez M. (2020). Lysosomal recycling of amino acids affects ER quality control. Sci. Adv..

[158] Zhu Y., Yan W., Tong L., Yang J., Ge S., Fan J., Jia R., Wen X. (2025). Metabolic Reprogramming: A Crucial Contributor to Anticancer Drug Resistance. MedComm.

[159] Paulusma C.C., Lamers W.H., Broer S., van de Graaf S.F.J. (2022). Amino acid metabolism, transport and signalling in the liver revisited. Biochem. Pharmacol..

[160] Hur W., Seong G.H., Choi H.S. (2025). Next generation drug clearance insights: Real-time tracking in hepatobiliary and renal systems. Light Sci. Appl..

[161] Knol M.G.E., Wulfmeyer V.C., Müller R.U., Rinschen M.M. (2024). Amino acid metabolism in kidney health and disease. Nat. Rev. Nephrol..

[162] Andritsos L.A., Dunavin N., Lozanski G., Jones J.A., Blachly J.S., Lucas D.M., Byrd J.C., Kraut E., Grever M.R. (2015). Reduced dose pentostatin for initial management of hairy cell leukemia patients who have active infection or risk of hemorrhage is safe and effective. Haematologica.

[163] Sanders T.J., Nabel C.S., Brouwer M., Hermant A.L., Chaible L., Deglasse J.P., Rosewick N., Pabois A., Cathou W., Smets A. (2025). Inhibition of ENT1 relieves intracellular adenosine-mediated T cell suppression in cancer. Nat. Immunol..

[164] Krawczyk A., Kravčenia B., Maślanka T. (2025). Mycophenolate mofetil: An update on its mechanism of action and effect on lymphoid tissue. Front. Immunol..

[165] Songwisit S., Kosiyakul P., Jitprapaikulsan J., Prayoonwiwat N., Ungprasert P., Siritho S. (2020). Efficacy and safety of mycophenolate mofetil therapy in neuromyelitis optica spectrum disorders: A systematic review and meta-analysis. Sci. Rep..

[166] Zhang C., Chu M. (2018). Leflunomide: A promising drug with good antitumor potential. Biochem. Biophys. Res. Commun..

[167] Mullen N.J., Shukla S.K., Thakur R., Kollala S.S., Wang D., Chaika N., Santana J.F., Miklavcic W.R., LaBreck D.A., Mallareddy J.R. (2024). DHODH inhibition enhances the efficacy of immune checkpoint blockade by increasing cancer cell antigen presentation. eLife.

[168] Kakkadath M., Naidu D., Kanthlal S.K., Sharun K. (2025). Combating Methotrexate Resistance in Cancer Treatment: A Review on Navigating Pathways and Enhancing Its Efficacy with Fat-Soluble Vitamins. Scientifica.

[169] Yang R., Wang B., Su Z., Song Y., Zhang Y., Liu Y., Zhang Y., He C., Yang X., Zhong F. (2025). Methotrexate exerts antitumor immune activity and improves the clinical efficacy of immunotherapy in patients with solid tumors. Sci. Transl. Med..

[170] Meng X.N., Ma J.F., Liu Y.H., Li S.Q., Wang X., Zhu J., Cai M.D., Zhang H.S., Song T., Xing S. (2024). Dynamic genomic changes in methotrexate-resistant human cancer cell lines beyond DHFR amplification suggest potential new targets for preventing drug resistance. Br. J. Cancer..

[171] Zhong C., Wang S., Jiang W.J., Li Z., Wang X., Fan S., Huang J., Wu H.J., Sheng R., Fei T. (2025). Chemoresistance mechanisms to 5-Fluorouracil and reversal strategies in lung and breast cancer. Sci. Rep..

[172] Biswas M., Sengupta S., Gandhi K.A., Gupta S.K., Gera P.B., Nayak B.S., Jagadeb M., Gota V., Sonawane A. (2025). Engineered L-asparaginase variants with enhanced therapeutic properties to improve treatment of childhood acute lymphatic leukemia. Cancer Gene Ther..

[173] Wang X., Gong W., Xiong X., Jia X., Xu J. (2024). Asparagine: A key metabolic junction in targeted tumor therapy. Pharmacol. Res..

[174] Montesinos P., Recher C., Vives S., Zarzycka E., Wang J., Bertani G., Heuser M., Calado R.T., Schuh A.C., Yeh S.P. (2022). Ivosidenib and Azacitidine in IDH1-Mutated Acute Myeloid Leukemia. N. Engl. J. Med..

[175] Reinbold R., Hvinden I.C., Rabe P., Herold R.A., Finch A., Wood J., Morgan M., Staudt M., Clifton I.J., Armstrong F.A. (2022). Resistance to the isocitrate dehydrogenase 1 mutant inhibitor ivosidenib can be overcome by alternative dimer-interface binding inhibitors. Nat. Commun..

[176] Venugopal S., Takahashi K., Daver N., Maiti A., Borthakur G., Loghavi S., Short N.J., Ohanian M., Masarova L., Issa G. (2022). Efficacy and safety of enasidenib and azacitidine combination in patients with IDH2 mutated acute myeloid leukemia and not eligible for intensive chemotherapy. Blood Cancer J..

[177] Richard-Carpentier G., Gupta G., Koraksic C., Cathelin S., Wang L., Bankar A., Davidson M., Gupta V., Maze D., Minden M.D. (2025). Enasidenib plus venetoclax in patients with IDH2-mutated relapsed or refractory acute myeloid leukaemia or myelodysplastic syndrome (ENAVEN-AML): A multicentre, single-arm, phase 1b/2 trial. Lancet Haematol..

[178] Yao K., Liu H., Yu S., Zhu H., Pan J. (2022). Resistance to mutant IDH inhibitors in acute myeloid leukemia: Molecular mechanisms and therapeutic strategies. Cancer Lett..

[179] Gouda M.A., Voss M.H., Tawbi H., Gordon M., Tykodi S.S., Lam E.T., Vaishampayan U., Tannir N.M., Chaves J., Nikolinakos P. (2025). A phase I/II study of the safety and efficacy of telaglenastat (CB-839) in combination with nivolumab in patients with metastatic melanoma, renal cell carcinoma, and non-small-cell lung cancer. ESMO Open..

[180] Gonçalves A.C., Richiardone E., Jorge J., Polónia B., Xavier C.P.R., Salaroglio I.C., Riganti C., Vasconcelos M.H., Corbet C., Sarmento-Ribeiro A.B. (2021). Impact of cancer metabolism on therapy resistance—Clinical implications. Drug Resist. Updat..

[181] Rouault C.D., Charafe-Jauffret E., Ginestier C. (2025). The interplay of DNA damage, epigenetics and tumour heterogeneity in driving cancer cell fitness. Nat. Commun..

[182] Fielden J., Siegner S.M., Gallagher D.N., Schröder M.S., Dello Stritto M.R., Lam S., Kobel L., Schlapansky M.F., Jackson S.P., Cejka P. (2025). Comprehensive interrogation of synthetic lethality in the DNA damage response. Nature.

[183] Olaizola I., Odriozola-Gimeno M., Olaizola P., Caballero-Camino F.J., Pastor-Toyos N., Tena-Garitaonaindia M., Lapitz A., Val B., Guimaraes A.R., Asensio M. (2025). New platinum derivatives selectively cause double-strand DNA breaks and death in naïve and cisplatin-resistant cholangiocarcinomas. J. Hepatol..

[184] Panieri E., Santoro M.M. (2016). ROS homeostasis and metabolism: A dangerous liason in cancer cells. Cell Death Dis..

[185] Wilsker D.F., Barrett A.M., Dull A.B., Lawrence S.M., Hollingshead M.G., Chen A., Kummar S., Parchment R.E., Doroshow J.H., Kinders R.J. (2019). Evaluation of Pharmacodynamic Responses to Cancer Therapeutic Agents Using DNA Damage Markers. Clin. Cancer Res..

[186] Verginadis I.I., Citrin D.E., Ky B., Feigenberg S.J., Georgakilas A.G., Hill-Kayser C.E., Koumenis C., Maity A., Bradley J.D., Lin A. (2025). Radiotherapy toxicities: Mechanisms, management, and future directions. Lancet.

[187] Wei W., Ren Y., Lan J., Yi J., Wang M., Zhang Y., Wang S., Xu Y., Han G., Fu Y. (2026). Ionizing radiation: Molecular mechanisms, biological effects, and therapeutic targets. Mol. Biomed..

[188] Huang R.X., Zhou P.K. (2020). DNA damage response signaling pathways and targets for radiotherapy sensitization in cancer. Signal Transduct. Target. Ther..

[189] Yu Z.W., Zheng M., Fan H.Y., Liang X.H., Tang Y.L. (2024). Ultraviolet (UV) radiation: A double-edged sword in cancer development and therapy. Mol. Biomed..

[190] Roos W.P., Thomas A.D., Kaina B. (2016). DNA damage and the balance between survival and death in cancer biology. Nat. Rev. Cancer.

[191] Andrezálová L., Országhová Z. (2021). Covalent and noncovalent interactions of coordination compounds with DNA: An overview. J. Inorg. Biochem..

[192] van de Kooij B., van der Wal F.J., Rother M.B., Creixell P., Stout M., Wiegant W., Joughin B.A., Vornberger J., van Vugt M.A.T.M., Altmeyer M. (2024). The Fanconi anemia core complex promotes CtIP-dependent end-resection to drive homologous recombination at DNA double-strand breaks. Nat. Commun..

[193] Su W.P., Ho Y.C., Wu C.K., Hsu S.H., Shiu J.L., Huang J.C., Chang S.B., Chiu W.T., Hung J.J., Liu T.L. (2017). Chronic treatment with cisplatin induces chemoresistance through the TIP60-mediated Fanconi anemia and homologous recombination repair pathways. Sci. Rep..

[194] Gutierrez R., O’Connor T.R. (2021). DNA direct reversal repair and alkylating agent drug resistance. Cancer Drug Resist..

[195] Fang Q. (2024). The Versatile Attributes of MGMT: Its Repair Mechanism, Crosstalk with Other DNA Repair Pathways, and Its Role in Cancer. Cancers.

[196] Venugopal S., Sharma V., Mehra A., Singh I., Singh G. (2022). DNA intercalators as anticancer agents. Chem. Biol. Drug Des..

[197] Sharma N.K., Bahot A., Sekar G., Bansode M., Khunteta K., Sonar P.V., Hebale A., Salokhe V., Sinha B.K. (2024). Understanding Cancer’s Defense against Topoisomerase-Active Drugs: A Comprehensive Review. Cancers.

[198] Liu T., Cai T., Huo J., Liu H., Li A., Yin M., Mei Y., Zhou Y., Fan S., Lu Y. (2024). Force-enhanced sensitive and specific detection of DNA-intercalative agents directly from microorganisms at single-molecule level. Nucleic Acids Res..

[199] Chaudhary P., Janmeda P., Docea A.O., Yeskaliyeva B., Abdull Razis A.F., Modu B., Calina D., Sharifi-Rad J. (2023). Oxidative stress, free radicals and antioxidants: Potential crosstalk in the pathophysiology of human diseases. Front. Chem..

[200] Hong Y., Boiti A., Vallone D., Foulkes N.S. (2024). Reactive Oxygen Species Signaling and Oxidative Stress: Transcriptional Regulation and Evolution. Antioxidants.

[201] Chandimali N., Bak S.G., Park E.H., Lim H.J., Won Y.S., Kim E.K., Park S.I., Lee S.J. (2025). Free radicals and their impact on health and antioxidant defenses: A review. Cell Death Discov..

[202] Suh M., Proctor D., Chappell G., Rager J., Thompson C., Borghoff S., Finch L., Ellis-Hutchings R., Wiench K. (2018). A review of the genotoxic, mutagenic, and carcinogenic potentials of several lower acrylates. Toxicology.

[203] Gollapudi B.B., Williams A.L., Bus J.S. (2021). A review of the genotoxicity of the industrial chemical cumene. Mutat. Res. Rev. Mutat. Res..

[204] Audebert M., Assmann A.S., Azqueta A., Babica P., Benfenati E., Bortoli S., Bouwman P., Braeuning A., Burgdorf T., Coumoul X. (2023). New approach methodologies to facilitate and improve the hazard assessment of non-genotoxic carcinogens-a PARC project. Front. Toxicol..

[205] Strupp C., Corvaro M., Cohen S.M., Corton J.C., Ogawa K., Richert L., Jacobs M.N. (2023). Increased Cell Proliferation as a Key Event in Chemical Carcinogenesis: Application in an Integrated Approach for the Testing and Assessment of Non-Genotoxic Carcinogenesis. Int. J. Mol. Sci..

[206] Gillespie L., Martin J.H., Anderson A.L., Bernstein I.R., Stanger S.J., Trigg N.A., Schjenken J.E., Gannon A.L., Parameswaran S., Smyth S.P. (2025). Exposure of mice to environmentally relevant per- and polyfluoroalkyl substances (PFAS) alters the sperm epigenome. Commun. Biol..

[207] Hasenböhler A., Javaux G., Payen de la Garanderie M., de Edelenyi F.S., Yvroud-Hoyos P., Agaësse C., De Sa A., Huybrechts I., Pierre F., Audebert M. (2026). Intake of food additive preservatives and incidence of cancer: Results from the NutriNet-Santé prospective cohort. BMJ.

[208] Szewczyk-Roszczenko O., Roszczenko P., Vassetzky Y., Sjakste N. (2025). Genotoxic consequences of viral infections. Npj Viruses.

[209] de Martel C., Georges D., Bray F., Ferlay J., Clifford G.M. (2020). Global burden of cancer attributable to infections in 2018: A worldwide incidence analysis. Lancet Glob Health.

[210] Jain U., Saxena K., Chauhan N. (2021). *Helicobacter pylori* induced reactive oxygen Species: A new and developing platform for detection. Helicobacter.

[211] Caceres I., Khoury A.A., Khoury R.E., Lorber S., Oswald I.P., Khoury A.E., Atoui A., Puel O., Bailly J.D. (2020). Aflatoxin Biosynthesis and Genetic Regulation: A Review. Toxins.

[212] Herr L.M., Schaffer E.D., Fuchs K.F., Datta A., Brosh R.M. (2024). Replication stress as a driver of cellular senescence and aging. Commun. Biol..

[213] Tang C., Livingston M.J., Safirstein R., Dong Z. (2023). Cisplatin nephrotoxicity: New insights and therapeutic implications. Nat. Rev. Nephrol..

[214] Patel H., Wu Z.X., Chen Y., Bo L., Chen Z.S. (2021). Drug resistance: From bacteria to cancer. Mol. Biomed..

[215] Ingham J., Ruan J.L., Coelho M.A. (2025). Breaking barriers: We need a multidisciplinary approach to tackle cancer drug resistance. BJC Rep..

[216] Mitchell E., Pham M.H., Clay A., Sanghvi R., Williams N., Pietsch S., Hsu J.I., Øbro N.F., Jung H., Vedi A. (2025). The long-term effects of chemotherapy on normal blood cells. Nat. Genet..

[217] Drew Y., Zenke F.T., Curtin N.J. (2025). DNA damage response inhibitors in cancer therapy: Lessons from the past, current status and future implications. Nat. Rev. Drug Discov..

[218] Groelly F.J., Fawkes M., Dagg R.A., Blackford A.N., Tarsounas M. (2023). Targeting DNA damage response pathways in cancer. Nat. Rev. Cancer.

[219] Li Q., Qian W., Zhang Y., Hu L., Chen S., Xia Y. (2023). A new wave of innovations within the DNA damage response. Signal Transduct. Target. Ther..

[220] Pyndiah S., Tanida S., Ahmed K.M., Cassimere E.K., Choe C., Sakamuro D. (2011). c-MYC suppresses BIN1 to release poly(ADP-ribose) polymerase 1: A mechanism by which cancer cells acquire cisplatin resistance. Sci. Signal..

[221] Folk W.P., Kumari A., Iwasaki T., Cassimere E.K., Pyndiah S., Martin E., Homlar K., Sakamuro D. (2021). New Synthetic Lethality Re-Sensitizing Platinum-Refractory Cancer Cells to Cisplatin In Vitro: The Rationale to Co-Use PARP and ATM Inhibitors. Int. J. Mol. Sci..

[222] Milano L., Gautam A., Caldecott K.W. (2024). DNA damage and transcription stress. Mol. Cell.

[223] Gu L., Li M., Li C.M., Haratipour P., Lingeman R., Jossart J., Gutova M., Flores L., Hyde C., Kenjić N. (2023). Small molecule targeting of transcription-replication conflict for selective chemotherapy. Cell Chem. Biol..

[224] Chiolo I., Altmeyer M., Legube G., Mekhail K. (2025). Nuclear and genome dynamics underlying DNA double-strand break repair. Nat. Rev. Mol. Cell Biol..

[225] Sanders J.T., Freeman T.F., Xu Y., Golloshi R., Stallard M.A., Hill A.M., San Martin R., Balajee A.S., McCord R.P. (2020). Radiation-induced DNA damage and repair effects on 3D genome organization. Nat. Commun..

[226] Vcelkova T., Reiter W., Zylka M., Hollenstein D.M., Schuckert S., Hartl M., Seiser C. (2023). GSE1 links the HDAC1/CoREST co-repressor complex to DNA damage. Nucleic Acids Res..

[227] Greenblatt J.F., Alberts B.M., Krogan N.J. (2024). Discovery and significance of protein-protein interactions in health and disease. Cell.

[228] Fu H., Mo X., Ivanov A.A. (2025). Decoding the functional impact of the cancer genome through protein-protein interactions. Nat. Rev. Cancer.

[229] Wang F., Braverman J., Eng G., Leylek O., Petrone N.L., Honeycutt D.S., Imada S., Pallares B., Zhang D., Mrosla J.M. (2025). Leveraging platinum-protein interactions to overcome chemoresistance. Nat. Commun..

[230] Yang X., Lan T., Zhang B., Tao X., Qi W., Xie K., Cai Y., Liu C., Han J., Wu H. (2025). Targeting ubiquitination in disease and therapy. Signal Transduct. Target. Ther..

[231] Magits W., Sablina A.A. (2022). The regulation of the protein interaction network by monoubiquitination. Curr. Opin. Struct. Biol..

[232] Wootton L.M., Morgan E.L. (2025). Ubiquitin and ubiquitin-like proteins in HPV-driven carcinogenesis. Oncogene.

[233] Chen X., Htet Z.M., López-Alfonzo E., Martin A., Walters K.J. (2021). Proteasome interaction with ubiquitinated substrates: From mechanisms to therapies. FEBS J..

[234] Collins G.A., Goldberg A.L. (2017). The Logic of the 26S Proteasome. Cell.

[235] Su R., Shao Y., Wang Q., Liu D., Wang Y., Kong D., Qiu Y. (2026). Targeting the ubiquitin-proteasome system and drug therapy in colorectal cancer. J. Mol. Cell Biol..

[236] Correa Marrero M., Mello V.H., Sartori P., Beltrao P. (2025). Global comparative structural analysis of responses to protein phosphorylation. Nat. Commun..

[237] Shyam Sunder S., Sharma U.C., Pokharel S. (2023). Adverse effects of tyrosine kinase inhibitors in cancer therapy: Pathophysiology, mechanisms and clinical management. Signal Transduct. Target. Ther..

[238] Kumar R., Goel H., Solanki R., Rawat L., Tabasum S., Tanwar P., Pal S., Sabarwal A. (2024). Recent developments in receptor tyrosine kinase inhibitors: A promising mainstay in targeted cancer therapy. Med. Drug Discov..

[239] Latosińska M., Latosińska J.N. (2024). Serine/Threonine Protein Kinases as Attractive Targets for Anti-Cancer Drugs-An Innovative Approach to Ligand Tuning Using Combined Quantum Chemical Calculations, Molecular Docking, Molecular Dynamic Simulations, and Network-like Similarity Graphs. Molecules.

[240] Gild M.L., Tsang V.H.M., Clifton-Bligh R.J., Robinson B.G. (2021). Multikinase inhibitors in thyroid cancer: Timing of targeted therapy. Nat. Rev. Endocrinol..

[241] Jing R., Wu N., Wu Y., Zhang Q., Liang Q., Huang P., Yi S. (2024). Efficacy and Safety of Multikinase Inhibitors for Patients with Refractory Thyroid Cancer: Systematic Review and Network Meta-Analysis. J. Clin. Endocrinol. Metab..

[242] Hayashi K., Sekaran S., Simpson P., Ebmeier C.C., Michel C.R., Ahn N.G. (2026). Variable thresholds for phosphorylation targets of the ERK signaling pathway. Proc. Natl. Acad. Sci. USA.

[243] Yang Y., Li S., Wang Y., Zhao Y., Li Q. (2022). Protein tyrosine kinase inhibitor resistance in malignant tumors: Molecular mechanisms and future perspective. Signal Transduct. Target. Ther..

[244] Raivola J., Rantanen F., Dini A., Piki E., Barker H., Multamäki E., Bentz F., Sunkel P., Kiiski A.M., Paavolainen L. (2026). ROR1-PI3K/AKT signaling drives adaptive resistance to cell cycle blockade in TP53 mutated ovarian cancer. Cell Death Dis..

[245] Gnanasundram S.V., Pyndiah S., Daskalogianni C., Armfield K., Nylander K., Wilson J.B., Fåhraeus R. (2017). PI3Kδ activates E2F1 synthesis in response to mRNA translation stress. Nat. Commun..

[246] Cooper A.J., Sequist L.V., Lin J.J. (2022). Third-generation EGFR and ALK inhibitors: Mechanisms of resistance and management. Nat. Rev. Clin. Oncol..

[247] Asciolla J.J., Wu X., Adamopoulos C., Gavathiotis E., Poulikakos P.I. (2025). Resistance mechanisms and therapeutic strategies of CDK4 and CDK6 kinase targeting in cancer. Nat. Cancer.

[248] Pandey K., An H.J., Kim S.K., Lee S.A., Kim S., Lim S.M., Kim G.M., Sohn J., Moon Y.W. (2019). Molecular mechanisms of resistance to CDK4/6 inhibitors in breast cancer: A review. Int. J. Cancer.

[249] Wright S.C.E., Vasilevski N., Serra V., Rodon J., Eichhorn P.J.A. (2021). Mechanisms of Resistance to PI3K Inhibitors in Cancer: Adaptive Responses, Drug Tolerance and Cellular Plasticity. Cancers.

[250] Hermansen J.U., Athanasiadis P., Yin Y., Rise A.F., Arribas A.J., Cascione L., Russnes H.G., Helland Å., Mato A.R., Bertoni F. (2025). Proteasome inhibition overcomes resistance to targeted therapies in B-cell malignancy models and in an index patient. Cell Death Dis..

[251] Bi J., Zhang Y., Malmrose P.K., Losh H.A., Newtson A.M., Devor E.J., Thiel K.W., Leslie K.K. (2022). Blocking autophagy overcomes resistance to dual histone deacetylase and proteasome inhibition in gynecologic cancer. Cell Death Dis..

[252] Lü S., Wang J. (2013). The resistance mechanisms of proteasome inhibitor bortezomib. Biomark. Res..

[253] Zuo W.F., Pang Q., Zhu X., Yang Q.Q., Zhao Q., He G., Han B., Huang W. (2024). Heat shock proteins as hallmarks of cancer: Insights from molecular mechanisms to therapeutic strategies. J. Hematol. Oncol..

[254] Zhang C., Li J., Tang Q., Li L., Cao D. (2025). Targeting proteostasis for cancer therapy: Current advances, challenges, and future perspectives. Mol. Cancer.

[255] Mweempwa A., Wilson M.K. (2019). Mechanisms of resistance to PARP inhibitors—An evolving challenge in oncology. Cancer Drug Resist..

[256] Jackson L.M., Moldovan G.L. (2022). Mechanisms of PARP1 inhibitor resistance and their implications for cancer treatment. NAR Cancer.

[257] Zhong Q., Xiao X., Qiu Y., Xu Z., Chen C., Chong B., Zhao X., Hai S., Li S., An Z. (2023). Protein posttranslational modifications in health and diseases: Functions, regulatory mechanisms, and therapeutic implications. MedComm.

[258] Ramazi S., Zahiri J. (2021). Posttranslational modifications in proteins: Resources, tools and prediction methods. Database.

[259] Lee J.M., Hammarén H.M., Savitski M.M., Baek S.H. (2023). Control of protein stability by post-translational modifications. Nat. Commun..

[260] Geffen Y., Anand S., Akiyama Y., Yaron T.M., Song Y., Johnson J.L., Govindan A., Babur Ö., Li Y., Huntsman E. (2023). Pan-cancer analysis of post-translational modifications reveals shared patterns of protein regulation. Cell.

[261] Li Z., Zhu T., Wu Y., Yu Y., Zang Y., Yu L., Zhang Z. (2025). Functions and mechanisms of non-histone post-translational modifications in cancer progression. Cell Death Discov..

[262] Pandey N., Franklin K.A., Haynes K.A., Rapé M., Cristea I.M. (2023). Adding post-translational modifications and protein-protein interactions to protein schematics. Trends Biochem. Sci..

[263] Gu J., He Y., He C., Zhang Q., Huang Q., Bai S., Wang R., You Q., Wang L. (2025). Advances in the structures, mechanisms and targeting of molecular chaperones. Signal Transduct. Target. Ther..

[264] Cai R.D., Lin M.J., Ye Q.M. (2025). Overcoming cancer drug resistance through small-molecule targeting of HSP90 and HSP70. Cancer Drug Resist..

[265] Cho W.C., Wong C.F. (2025). Potential benefits of combined treatment with Hsp90 inhibitor AUY922 and cisplatin for overcoming drug resistance in nasopharyngeal carcinoma. Am. J. Cancer Res..

[266] Fog C.K., Zago P., Malini E., Solanko L.M., Peruzzo P., Bornaes C., Magnoni R., Mehmedbasic A., Petersen N.H.T., Bembi B. (2018). The heat shock protein amplifier arimoclomol improves refolding, maturation and lysosomal activity of glucocerebrosidase. EBioMedicine.

[267] Kunachowicz D., Król-Kulikowska M., Raczycka W., Sleziak J., Błażejewska M., Kulbacka J. (2024). Heat Shock Proteins, a Double-Edged Sword: Significance in Cancer Progression, Chemotherapy Resistance and Novel Therapeutic Perspectives. Cancers.

[268] Alderson T.R., Roche J., Gastall H.Y., Dias D.M., Pritišanac I., Ying J., Bax A., Benesch J.L.P., Baldwin A.J. (2019). Local unfolding of the HSP27 monomer regulates chaperone activity. Nat. Commun..

[269] Selig E.E., Zlatic C.O., Cox D., Mok Y.F., Gooley P.R., Ecroyd H., Griffin M.D.W. (2020). N- and C-terminal regions of αB-crystallin and Hsp27 mediate inhibition of amyloid nucleation, fibril binding, and fibril disaggregation. J. Biol. Chem..

[270] Nada H., Choi Y., Kim S., Jeong K.S., Meanwell N.A., Lee K. (2024). New insights into protein-protein interaction modulators in drug discovery and therapeutic advance. Signal Transduct. Target. Ther..

[271] Fang Y., Cheng L., Huang M., Cao Y., Zou Q., Cai J., Zhang Y., Xia Y., Huang H., Chen X. (2025). Heat shock factor 1 promotes proliferation and chemoresistance in diffuse large B-cell lymphoma by enhancing the cell cycle and DNA repair. Cell Death Dis..

[272] Wang B., Zhang L., Dai T., Qin Z., Lu H., Zhang L., Zhou F. (2021). Liquid-liquid phase separation in human health and diseases. Signal Transduct. Target. Ther..

[273] Li Y., Liu Y., Yu X.Y., Xu Y., Pan X., Sun Y., Wang Y., Song Y.H., Shen Z. (2024). Membraneless organelles in health and disease: Exploring the molecular basis, physiological roles and pathological implications. Signal Transduct. Target. Ther..

[274] Wang F., Zhang Y. (2024). Physiology and pharmacological targeting of phase separation. J. Biomed. Sci..

[275] Moses D., Ginell G.M., Holehouse A.S., Sukenik S. (2023). Intrinsically disordered regions are poised to act as sensors of cellular chemistry. Trends Biochem. Sci..

[276] Wang Y.L., Zhao W.W., Shi J., Wan X.B., Zheng J., Fan X.J. (2023). Liquid-liquid phase separation in DNA double-strand breaks repair. Cell Death Dis..

[277] Hsiao A.S. (2024). Protein Disorder in Plant Stress Adaptation: From Late Embryogenesis Abundant to Other Intrinsically Disordered Proteins. Int. J. Mol. Sci..

[278] He Y., Bao X., Chen T., Jiang Q., Zhang L., He L.N., Zheng J., Zhao A., Ren J., Zuo Z. (2025). RPS 2.0: An updated database of RNAs involved in liquid-liquid phase separation. Nucleic Acids Res..

[279] Xu F., Ovalle M., Fu Y., Stuart M.A.C., Feringa B.L. (2025). Molecular motor-driven reversible liquid-liquid phase separation of supramolecular assemblies. Nat. Commun..

[280] Trivedi R., Nagarajaram H.A. (2022). Intrinsically Disordered Proteins: An Overview. Int. J. Mol. Sci..

[281] Pesce F., Bremer A., Tesei G., Hopkins J.B., Grace C.R., Mittag T., Lindorff-Larsen K. (2024). Design of intrinsically disordered protein variants with diverse structural properties. Sci. Adv..

[282] Liu C., Wu K., Choi H., Han H.L., Zhang X., Watson J.L., Ahn G., Zhang J.Z., Shijo S., Good L.L. (2025). Diffusing protein binders to intrinsically disordered proteins. Nature.

